# Direct Measurements of Isoprene Autoxidation: Pinpointing Atmospheric Oxidation in Tropical Forests

**DOI:** 10.1021/jacsau.1c00525

**Published:** 2022-03-18

**Authors:** Diogo J. Medeiros, Mark A. Blitz, Paul W. Seakins, Lisa K. Whalley

**Affiliations:** † School of Chemistry, University of Leeds, Leeds LS2 9JT, UK; ‡ National Centre for Atmospheric Science (NCAS), University of Leeds, Leeds LS2 9JT, UK

**Keywords:** kinetics, OH radical, isoprene, OH recycling, atmospheric chemistry, modeling

## Abstract

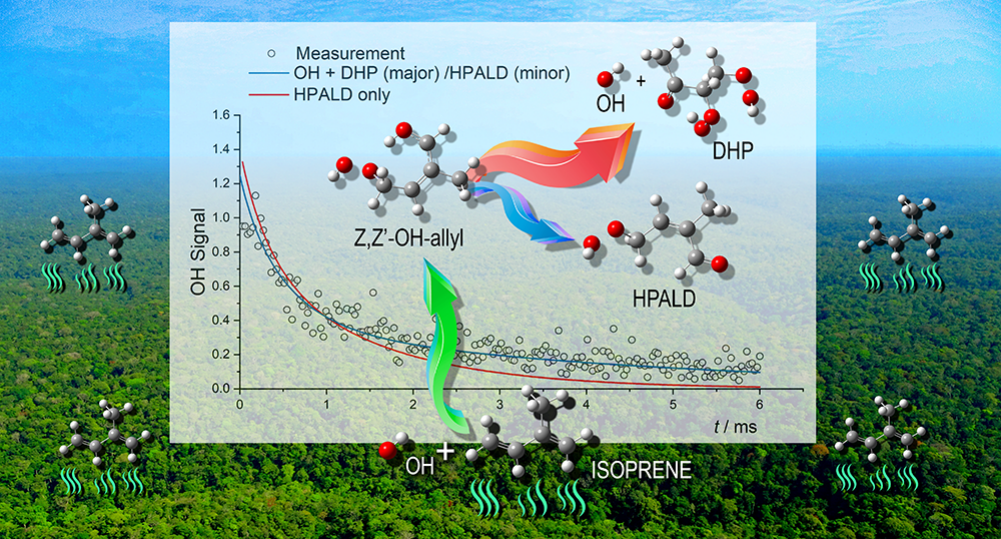

2-Methyl-1,3-butadiene (isoprene), released from biogenic sources, accounts for approximately a third of hydrocarbon emissions and is mainly removed by hydroxyl radicals, OH, the primary initiator of atmospheric oxidation. *In situ* measurements in clean tropical forests (high isoprene and low NO_*x*_) have measured OH concentrations up to an order of magnitude higher than model predictions, which impacts our understanding of global oxidation. In this study, direct, laser flash photolysis, laser-induced fluorescence measurements at elevated temperatures have observed OH recycling in the presence of isoprene and oxygen under conditions where interference from secondary or heterogeneous chemistry is minimal. Our results provide the first direct, time-resolved, experimental validation of the theory-based Leuven Isoprene Mechanism (LIM1), based on isomerization of isoprene-RO_2_ radicals and OH regeneration, that partially accounts for model:measurement divergence in OH. While our data can be fit with only minor alterations in important LIM1 parameters, and the overall rate of product formation is similar to LIM1, there are differences with the recent experimental study by Teng *et al*. *J. Am. Chem. Soc.***2017**, *139*, 5367–5377. In addition, our study indicates that the dihydroperoxide products are significantly enhanced over previous estimates. Dihydroperoxides are chemical and photochemical sources of OH, and the implications of enhanced hydroperoxide formation on the agreement between models and observations in tropical forests are examined.

## Introduction

The reactive hydrocarbon 2-methyl-1,3-butadiene (C_5_H_8_, isoprene) is the dominant biogenic emission (∼500 Tg yr^1^),^1,2^ accounting for approximately a third of hydrocarbon (RH) emissions, with tropical forests being strong sources. Isoprene released from biomass is oxidized in hours, mainly *via* its fast addition reaction^3^ with the hydroxyl radical, OH, followed by O_2_ addition, to form peroxy radicals, RO_2_. Isoprene oxidation leads to a rich array of oxygenated compounds,^4−8^ and a number of these products can lead to particle formation or growth.^9,10^ OH is the main atmospheric oxidant, controlling the atmospheric removal of methane and production of tropospheric ozone from hydrocarbon oxidation. Understanding global atmospheric oxidation is therefore vital for modeling future air quality and climate.

In urban environments, peroxy radicals are recycled back to OH *via* the HO_*x*_ cycle (simplified below):
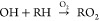

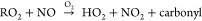

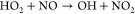


This HO_*x*_ cycle in a NO_*x*_ (NO + NO_2_)-rich environment is well established,^11,12^ and the rates for each step in the process are known to such an extent that chemical models of urban environments make reliable estimates of the observed levels of the OH concentration.^11^

In pristine tropical forests, the NO_*x*_ levels are considerably lower and RO_2_ radicals are predominantly removed by reaction with HO_2_ or other RO_2_. While some of these reactions can lead to OH (see below), the majority do not and hence the ability to recycle OH should be much reduced. With high isoprene concentrations (typically 3–10 ppbv),^13^ OH removal is rapid *via*R1, and with reduced recycling, predicted OH concentrations are low.
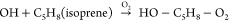
R1

Overall, this implies a low oxidation capacity for forested equatorial regions with implications on the rate of methane removal. However, this expectation was turned on its head when aircraft measurements in 2008 of [OH] above the Amazon were a factor of 12 higher than expected.^14,15^ Similarly, high [OH] measurements have been observed in other later campaigns, where the common factor is that the environment is low in NO_*x*_ and is dominated by isoprene chemistry.^16−18^ Studies of isoprene oxidation in simulation chambers (e.g., Fuchs *et al.*(19)) have confirmed significant OH regeneration.

These observations have provoked much speculation on the mechanism of the fast OH recycling. Under low NO_*x*_ conditions, RO_2_ chemistry, *via* either self-reaction or reaction with HO_2_, becomes dominant. While it is known that some reactions between RO_2_ and HO_2_ have a significant channel to OH,^20^ these and other alternatives, such as an epoxide channel,^9^ are insufficient to account for the OH measurements.

**Scheme 1:**
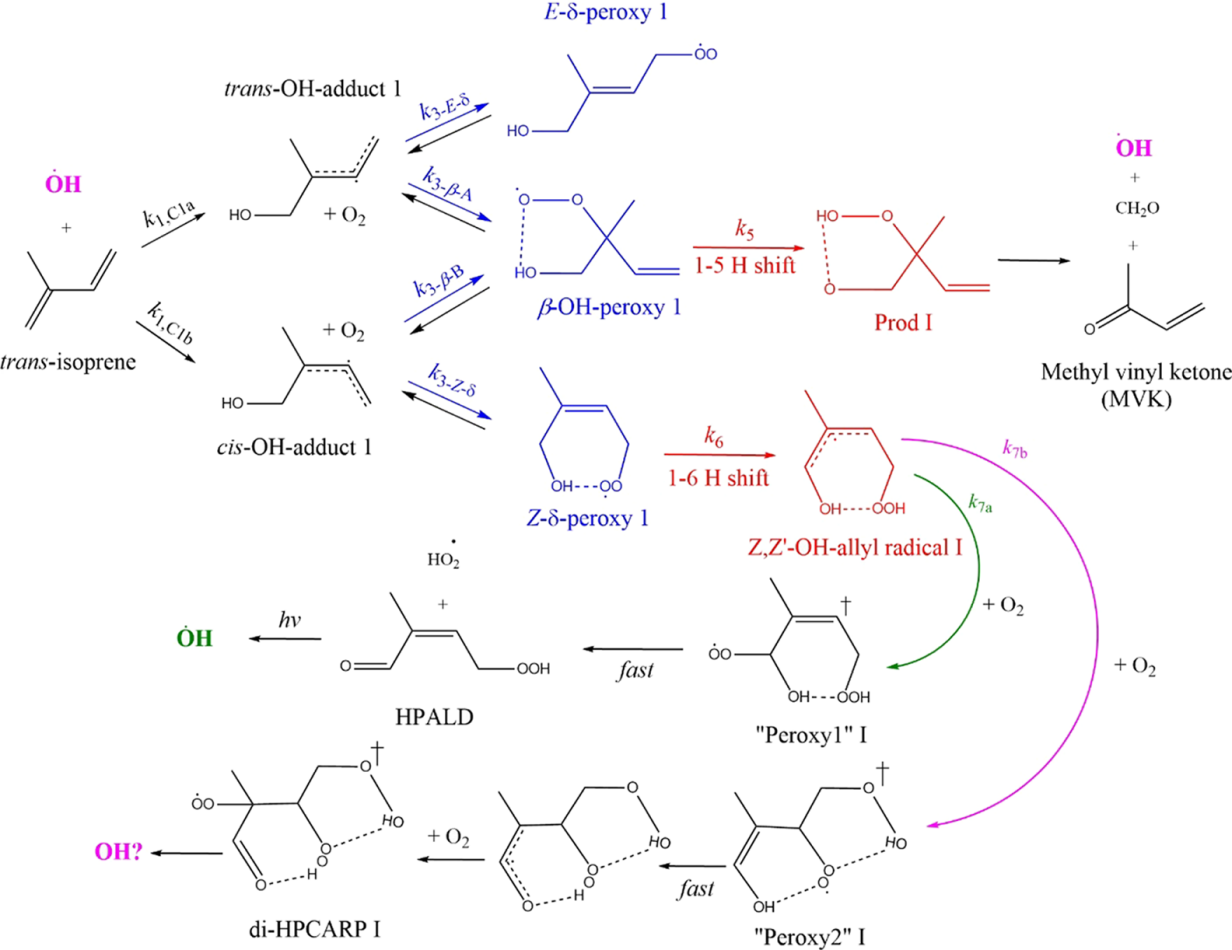
Leuven Isoprene Mechanism 1 as Proposed by Peeters *et al*.^7,8^ Case I, OH addition to carbon 1 of the primary chain. At 298 K, the peroxy radicals interconvert on the second timescale, but effective product formation is only *via* the 1–6 H shift, *k*_6_, when the highest energy peroxy isomer, *Z*-δ-peroxy, is populated. Overall, the timescale for product formation, HPALD and diHPCARP, is ∼100 s.

An explanation of this enhanced OH concentration, the Leuven Isoprene Mechanism, was proposed by Peeters *et al.*(7) (LIM0), where using theoretical calculations, it was shown that the OH/isoprene peroxy radical is relatively unstable and can isomerize to a number of channels, as summarized in Scheme 1. The long lifetime (10–1000 s) for RO_2_ removal in pristine forested conditions allows for isomerization between the various RO_2_ isomers, including the least stable *Z*-δ-peroxy radical that can lead to OH recycling. Scheme 1 shows the three peroxy radicals formed following OH addition to the substituted double bond at the C_1_ position; the analogous mechanism for OH addition at the C_4_ position is shown in the Supporting Information, Scheme S1.

The barrier for the 1–5 H shift to form OH + methylvinyl ketone (MVK) from the β-OH-peroxy (R5 in Scheme 1 or OH + methacrolein (MACR); Figure S1) is ∼10 kJ mol^–1^ higher than that for the 1–6 H shift from the *Z*-δ-peroxy to the *Z*,*Z*-OH-allyl radical (R6 in Scheme 1).^7,8,21^ The *Z*-δ-peroxy species is the precursor for the formation of hydroperoxy aldehyde, HPALD, or the dihydroperoxy aldehyde, diHPCARP, species that either directly lead to OH recycling (diHPCARP) or produce OH following photolysis (HPALD). The key to the LIM is the recognition of interconversion between RO_2_ isomers, allowing the least stable *Z*-δ-peroxy radical, which has the fastest route to OH production, to make a significant contribution to RO_2_ loss. Such isomerizations are not just limited to isoprene chemistry and, more recently, have been invoked in the formation of highly oxygenated multifunctional species (HOMS) from a range of VOCs. HOMS can have a significant impact on particle formation and growth.^22,23^

In the original paper by Peeters *et al.*,^7^ HPALD was considered to be the exclusive product and was calculated to form in ∼10 s. As HPALD is a conjugated hydroperoxide, it is reasonable to expect HPALD to be an effective atmospheric photolytic source of OH;^24^ recent experiments have verified that this is indeed the case.^25^ Therefore, the original work by Peeters *et al.* provided a rationale for the high [OH] observed over the Amazon.^15^

The LIM0 solution to explain high [OH] in isoprene-emitting forests was based on theoretical calculations, but it was shown to be problematic by Crounse *et al.*(6) when OH-initiated oxidation of isoprene was investigated in a simulation chamber. These experiments measured the rate of formation of HPALD to be ∼50 times slower than the bulk RO_2_ 1–6 H-shift rate indicated by Peeters *et al.*(7) and implied that there was still a mystery as to the source of OH.

However, in a follow-up study, Peeters *et al*.^8^ (LIM1), using a higher level of theory and considering the system in more detail, obtained general agreement, within a factor of 2, with the chamber study of Crounse *et al.*(6) LIM1 highlighted that the peroxy radical isomer that leads to HPALD occurs *via* an allylic intermediate that adds O_2_ in two ways: one way leads to HPALD + HO_2_ and the other way, following a further O_2_ addition, leads to a dihydroperoxy carbonyl peroxy radical (diHPCARP) (see Scheme 1 and R7)R7b.

More recently, the study by Teng *et al*.^4^ indicated that the overall kinetics for the RO_2_ radicals to form products *via* the 1–6 H shift (see Scheme 1) is considerably slower than given by LIM1. Also, the study by Berndt *et al*.^26^ indicated that the dominant product *via* the 1–6 H shift is hydroperoxy aldehyde, HPALD (see Scheme 1) rather than diHPCARP. The latest study by Novelli *et al.*(27) identified the OH recycling time but was unable to assign the HPALD yield.

In this paper, we report laboratory experiments where OH is generated by a laser photolysis pulse at time *t* = 0, and the [OH] is directly monitored in a time-resolved fashion by laser-induced fluorescence (LIF). The direct, *in situ*, time-resolved experiments were carried out at high temperatures (*T* = 420–583 K) so that OH recycling occurs on the millisecond timescale; this fast recycling avoids the possibility of interference from secondary chemistry or heterogeneous processes. We find that our OH traces, recorded over a wide temperature and pressure range, are fully described by LIM1 with remarkably little adjustment to the energy barriers and indicate that the main product *via* the 1–6 H shift forms the dihydroperoxy-carbonyl peroxy radical, diHPCARP (see Scheme 1). We use these slightly modified LIM1 parameters to assess the role of the LIM1 mechanism in OH production under conditions relevant to the OP3 campaign in Borneo.^28^

## Methods

### Experimental Section

The experiments were carried out in two distinctly different reaction cells: low-pressure^29,30^ and high-pressure reactors,^31,32^ in both cases using laser flash photolysis with hydroxyl radical, OH, detection by laser-induced fluorescence (LIF). The main difference between the cells is how the OH is measured. In the low-pressure cell (≤200 Torr), the OH detection is *in situ*. In the high-pressure cell (∼1400 Torr), a pinhole samples the OH (in about 20 μs) before LIF detection. The OH is detected within 1 cm of the pinhole, where the gas is jetting, i.e., undergoing relatively few collisions, and ensures that the kinetic traces are essentially unperturbed, i.e., identical to the kinetics in the low-pressure *in situ* OH cell.^3,33^ More details about this recently constructed high-pressure apparatus are given in the Supporting Information, Section S3.

The OH precursor, H_2_O_2_, was flashed with either a 248 nm KrF excimer laser or a 266 nm Nd:YAG laser to generate an instant OH concentration (typically [OH]_0_ < 1 × 10^12^ molecule cm^–3^ generated from ∼2 × 10^14^ to 7 × 10^14^ molecule cm^–3^ H_2_O_2_)
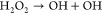


**Figure 1:**
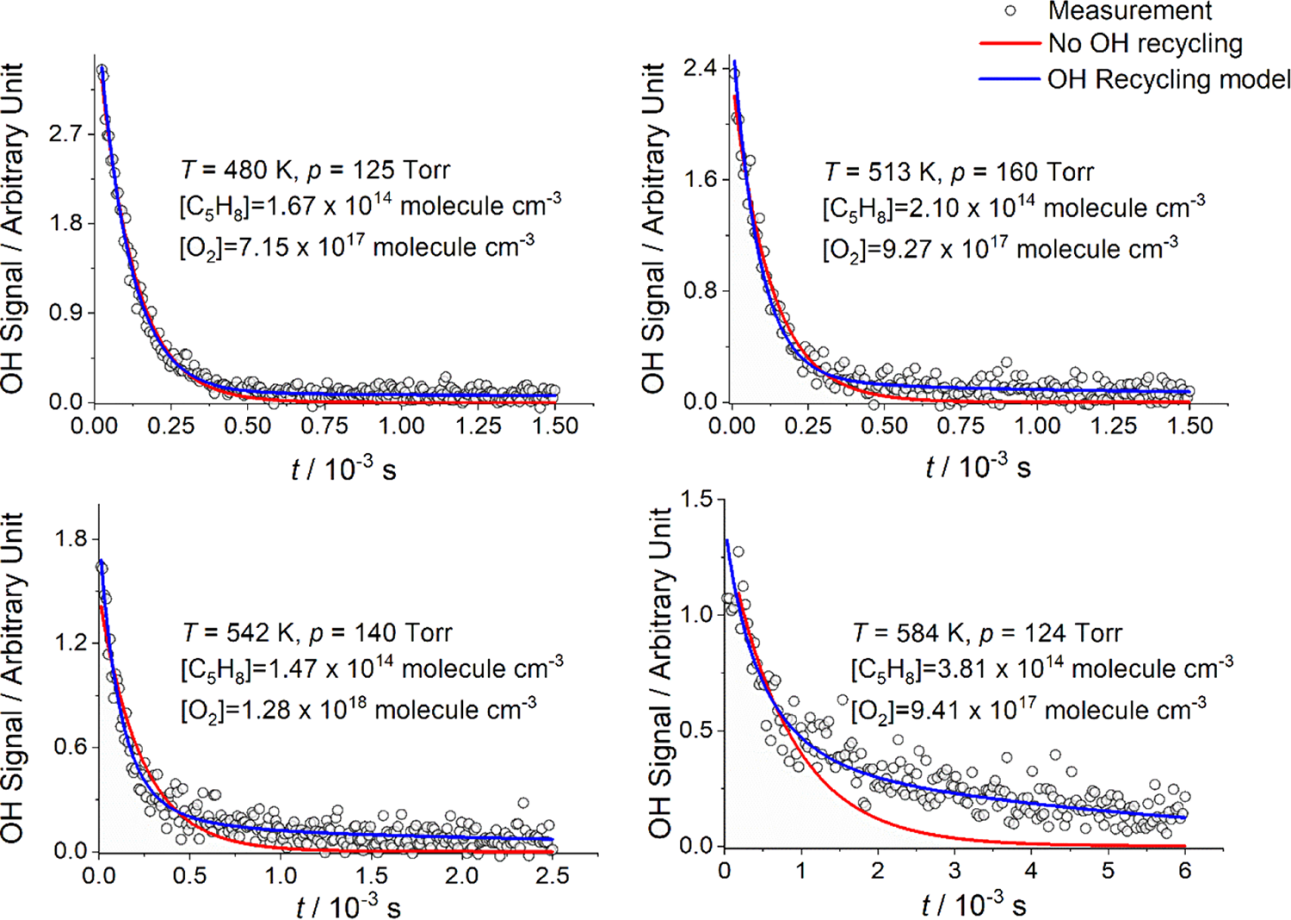
Typical OH traces showing increasing amounts of recycling. The red line is the single-exponential return to the baseline (no recycling), and the blue line is a fit to the data using a recycling model. The temperature (K), pressure (Torr), [C_5_H_8_], and [O_2_] (molecule cm^–3^) are given in the figure. The single exponential (red line) is based on our results when no O_2_ was added, i.e., no recycling.^3^ Above 420 K, all the traces are above the red line. This is evidence for recycling (blue), and in general, the traces show that the greater the recycling, the higher the temperature.

A dye laser was used to probe the OH concentration *via* LIF, where this second laser was wavelength-tuned to a feature of the hydroxyl radical spectrum, and ∼282 and ∼308 nm were used for the high- and low-pressure experiments, respectively (see the Supporting Information). The fluorescence photons passed through a 308 nm filter before being detected by a photomultiplier situated at right angles to the probe and photolysis lasers. By scanning the photolysis and probe lasers as a function of time, an OH time trace was recorded on a millisecond timescale (see Figure 1 for example). A typical trace consisted of 200 points, where each point was a result of averaging between 3 and 12 samples. The traces were usually recorded at 10 Hz, but a number of experiments carried out at rates down to 1 Hz confirmed that the effects of product buildup were insignificant.

The gases C_5_H_8_ (diluted with N_2_), O_2_, and N_2_ (buffering gas) were delivered to the reaction cell using calibrated mass flow controllers, and the total pressure was regulated using a valve in front of the exhaust pump. In the low-pressure cell, the pressure was between 100 and 200 Torr (13,332 and 26,664 Pa), as high as possible without significantly compromising the OH LIF signal. In the high-pressure cell, the total pressure (1350–1450 Torr, 180,000–193,300 Pa) and flow (∼10 SLM) were relatively constant to ensure that the temperature is known. The experimental conditions for these experiments are given in Table S2.

### Data Analysis (MATLAB)

The kinetics measured in this study are fully described by the reactions depicted in Scheme 1 and Scheme S1, plus the loss of OH in the absence of isoprene (<10% of the OH loss in the high-pressure system, predominantly due to the reaction with H_2_O_2_ and <3% in the more sensitive low-pressure cell; the enhanced sensitivity means that lower [H_2_O_2_] can be used) and the direct abstraction from isoprene by OH, which becomes significant at the temperatures of the present experiments (∼10% at 500 K) but which has been well characterized in our previous work.^3^ LIM1 is a fundamental description of the system, where *ab initio* structure calculations were undertaken to map out the potential energy surface of the reaction (the mechanism) and reaction rate theory was employed to calculate the rate coefficients. The rate coefficient expressions for the LIM1 reactions (32) and two additional reactions are listed in Table S1. Further reaction rate theory calculations were carried out to demonstrate that the system is independent of pressure (see Section S4 in the Supporting Information).

The program MATLAB^34^ has the capability to suitably adjust the parameters of this LIM1 mechanism and then numerically integrate it to best fit to the OH time traces. To improve parameter retrieval, data analysis was carried out globally, simultaneously fitting parameters to the 94 OH time-dependent traces.^35^ This approach is required as the OH trace data are described by many rate coefficients, and one trace alone will not guarantee a consistent and reliable extraction of temperature-dependent information. Global analysis is a technique that takes advantage of the relationships that exist in the data to better describe and identify the parameters of the system. To carry out the global analysis, the software package MATLAB R2016^34^ required a script to define LIM1 and adjust/impose constraints on the selected parameters during the global procedure. The ordinary differential equations of LIM1 were numerically integrated for the experimental conditions (*T*, [isoprene], [O_2_], and *k*_loss_) of each one of the 94 traces with the aid of the MATLAB ODE suite.^36^ Floatable parameters were adjusted following the trust region reflective algorithm.^37^ The objective function was defined as the sum of squared residuals (χ^2^) calculated from a comparison between experimental measurements and their corresponding numerical simulation. Each trace was appropriately weighted using the χ^2^ from fitting it individually using a flexible function, a bi-exponential. This individual fit χ^2^ represents a good approximation to the best fit so that, in the global analysis, the best value for χ^2^ divided by the number of traces, *n*_traces_, is 1.0. From Table 1, it can be seen that χ^2^/*n*_traces_ is within ∼20% of 1.0, and all the fits are shown in the Supporting Information, Section S7.

**Table 1:** Best-Fit Parameters from This Study and Comparison with the Literature^e^

parameter	this work (LIM1-Leeds) scenario 1	this work (LIM1-Leeds) scenario 14	Teng *et al*.^4^	Peeters *et al.*(8) (LIM1)
C_5_H_8_-OH + O_2_, *k*_3_, scaling factor				
*S*_3,*Z*-δ-RO2_	2.9 ± 1.1	11.7 ± 8.0	0.26^a^	1.0
*S*_3,other-RO2_	2.9 ± 1.1	4.8 ± 2.4	1.29^a^	1.0
*E*_–3,adjust_/kJ mol^–1^	0	–0.3 ± 2.0	–3.7^b^	0
1–5 H-shift barrier/kJ mol^–1^ (R5)	81.03	85.5 ± 1.9	81.03	81.03
1–6 H-shift barrier/kJ mol^–1^ (R6)	71.42	74.2 ± 2.8	72.37	71.42
*BFk*_7a_/*k*_7_(298 K)	0.25	0.19 ± 0.04		0.50
χ^2^/*n*_traces_	1.15	1.13		
*k*(bulk) s^–1^	0.0082	0.0076^c^	0.002^d^	0.008^d^

a The value is an average as each individual isomer was adjusted.

b The value is the average of all isomers.

c Defined as ln(2) divided by the time for half of products to form.

d Defined using the LIM1 definition.^8^ Both definitions of *k*(bulk) are similar at 298 K.

e Errors quoted at 2σ. Parameters from the other scenarios are given in the Supporting Information.

To test the LIM1 mechanism, the starting point was to adjust the minimum number of parameters and then incrementally float more and more parameters (the scenarios in the Supporting Information) to observe how well the parameters are defined and their deviation from LIM1. These adjusted parameters are color-highlighted in Table S1. In the results and discussion below, the components of LIM1 and how the data analysis links these components together, where appropriate, are described. This means that the kinetics of LIM1 are extensively tested, but even in the most flexible model, some of the rate coefficients are suitably constrained or linked.

## Results

At room temperature, OH decays in the presence of isoprene and oxygen returns to the baseline exponentially, which is consistent with the reaction to form RO_2_ (R1). As the temperature is increased, >420 K, it can be seen that the OH does not return exponentially (red lines in Figure 1) to the baseline and this is evidence that the system is recycling OH, as summarized by the overall reaction:
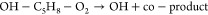
R2

However, the kinetics of the system are more complicated than just R2 as multiple RO_2_ isomers are present, and only two of the six RO_2_ isomers lead to HPALD/diHPCARP (see Scheme 1 and Scheme S1).

To match the LIM1 mechanism to our data, the mechanism should be adjusted logically and with the minimum number of parameter changes. Fortunately, some rate parameters in LIM1 are known and can be fixed: for example, the removal rate coefficient of OH with isoprene in the absence of O_2_ is exceptionally well known,^3^ so this can be fixed. The addition rate coefficients of O_2_ to the isomer adducts:

R3,_*i*=1–3_:
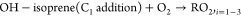
R3a

R3,_*i*=4–6_:
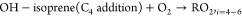
R3bare calculated in the LIM1 model. However, the crucial isomers are those forming *Z*-δ-peroxy (RO_2_,_*i*=3_ and RO_2_,_*i*=6_). Therefore, in our analysis, the RO_2_ isomers were split into two groups, reactions forming the δ RO_2_ (for either addition site) and those forming the other RO_2_ species (again for both addition sites):

R3,*Z*-δ-RO_2_:
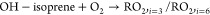
R3c

R3,other-RO_2_:
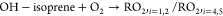
R3d

These R3,_*i*_ rate coefficients were initially assigned the values of the theoretical LIM1 model but were then adjusted *via* an additional temperature-independent scaling factor, *S*:
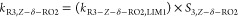
E1a
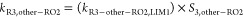
E1b

This adjustment means that, within the isomer split, the ratio of *k*_R3,*i*_ rate coefficients maintains the LIM1 ratio, which is expected to be correct, but allows for the larger uncertainty in the absolute *k*_R3,*i*_ values. In the various scenarios described in the Supporting Information, either a single scaling factor, *S*_3_, was used for both RO_2_ groups or the scaling factors shown in E1a and E1b could be varied independently.

The isomers *i* = 1–3 (formed from OH addition at C_1_) and *i* = 4–6 (OH addition at C_4_) cannot interconvert. However, within their set, they interconvert *via* their forward and reverse reactions, R_3_/R_–3_; see the LIM1 mechanism (Scheme 1).

R3,_*i*=1–3_:
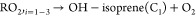
R3e

R3,_*i*=4–6_:
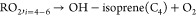
R3f

The reverse reactions are largely controlled by their binding energies, which again have been calculated by Peeters *et al.* In the system, the RO_2_ isomers equilibrate, and at our experimental temperatures, equilibrium is established rapidly. In our analysis, the redissociation rate coefficients, *k*_–3_, were initially assigned the LIM1 values and were adjusted using one parameter, *E*_–3,adjust_:
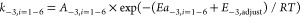
E2so that the binding energy of the RO_2_,_*i*=1,6_ maintained the LIM1 difference.

The RO_2_,_*i*_ species generally react to products:
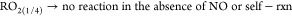
R4
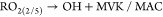
R5
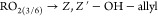
R6
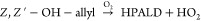
R7a
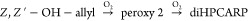
R7b

On the timescales of the chemistry in this study and at the low radical concentrations used, RO_2(1/4)_ is essentially unreactive. This is a significant advantage of our approach; for example, if the *E*-δ peroxy radicals are at 10% of the initial [OH] (say 1 × 10^11^ molecule cm^–3^) and undergo self-reactions with a high rate coefficient of 1 × 10^–10^ cm^3^ molecule^–1^ s^–1^, then the timescale of the loss process, 0.1 s, is ∼100 times slower than OH removal. R5 is a direct channel to OH but is slower than R6 due to its higher barrier (81.0 kJ mol^–1^ vs 71.4 kJ mol^–1^ in LIM1). At room temperature, loss of RO_2_,_*i*=1,6_*via*R5 and R6 has a half-life (*k*(bulk) = ln(2)/half-life)) of a few hundred seconds. Overall, HPALD and diHPCARP are the major products at room temperature, even though the peroxy radicals mainly exist as RO_2,2/5_. The rate coefficients for R5 and R6 were initially assigned their LIM1 values:
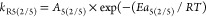
E3
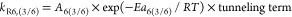
E4

In the Supporting Information, the starting models (scenarios 1–5) have the barrier values of LIM1, and then, the barriers are adjusted in unison (scenarios 6, 7, 12, and 13), where the energy gap between the isomers remains the same as the LIM1, and then, scenarios 8–11 and 14 have *Ea*_5_ and *Ea*_6_ adjusted independently. The 1,6 H-shift reaction rate coefficient of R6, *k*_R6,(3/6)_, is enhanced significantly at 298 K by quantum mechanical tunneling, hence the tunneling term in E4. However, at the temperatures of our experiments, this tunneling term is converging toward one (see the Supporting Information, Section S4 and Figure S3), so the considerable uncertainty associated with such calculations^8^ should not significantly impact our kinetic analysis.

**Figure 2:**
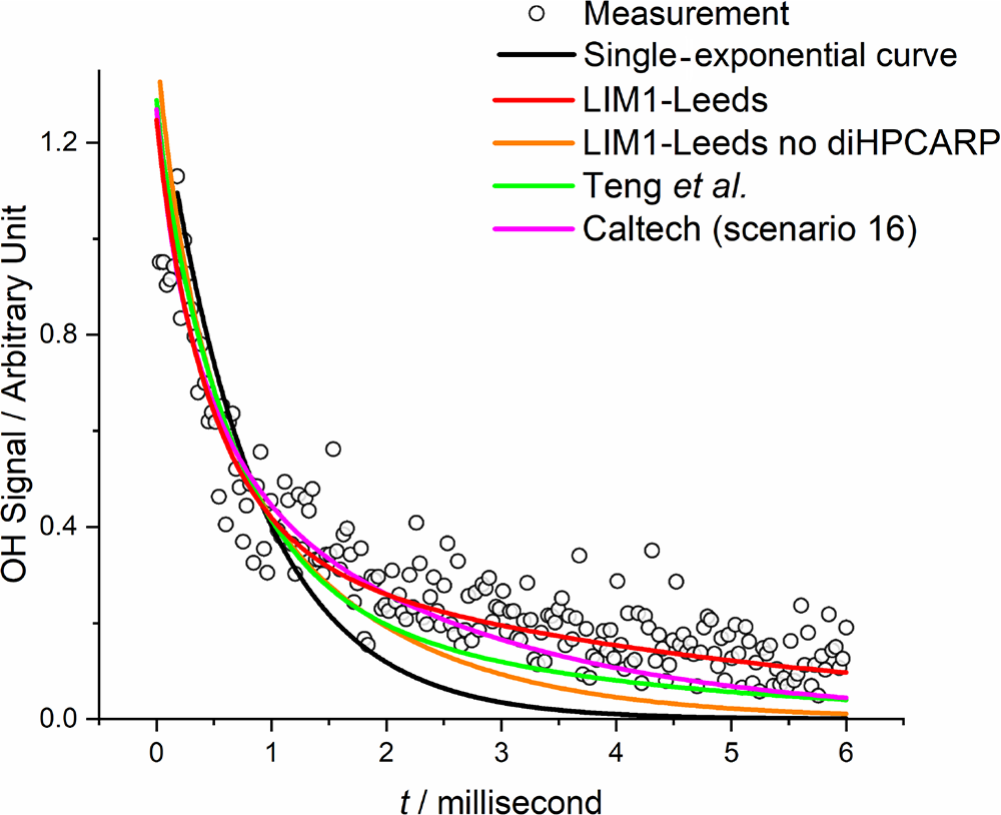
Comparison of the various models implemented when fitting a trace generated at 584 K and 124 Torr of N_2_, where [C_5_H_8_] and [O_2_] are equal to 3.81 × 10^14^ and 9.41 × 10^17^ molecules cm^–3^, respectively. The data are distinctly non-single exponential and are best fitted by LIM1-Leeds (scenario 3), where the diHPCARP recycles OH. The Caltech model^5^ (scenario 16 in the Supporting Information) is a refinement of the Teng *et al.* model, but neither provides a good description of the data.

Initially, when the data analysis only included R5 (not R6) as the only OH-producing channel, it was evident that the fit to the data was poor based on χ^2^ and visual inspection of the traces (see the LIM1-Leeds no diHPCARP fit to the data in Figure 2). This problem was overcome when it was recognized that diHPCARP, formed from the isomerization of the Ζ-δ-peroxy radical, decomposes to OH. Fast decomposition of diHPCARP to OH (R8, 0.1 s^–1^ at 298 K) was originally suggested and calculated by Peeters *et al*.^8^ and recently calculated to be even faster in the study by Novelli *et al*.^27^ Hence, R8 is essentially instantaneous under our experimental recycling temperatures, and therefore, our data analysis includes OH formation *via*R6, R7a, and R7b and the fast reaction:
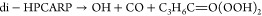
R8where C_3_H_6_C=O(OOH)_2_ is a dihydroperoxide carbonyl, DHP. The O_2_ reactions of R7a and R7b are assumed to have the same rate coefficients as R3 (*k*_R7_ is explored further in the Supporting Information, Section S5). This fast source of OH occurs at the expense of HPALD formation and removes the contradiction of the early chamber experiments that modeled HPALD formation, assuming that the Ζ-δ-peroxy radical isomerization only produced HPALD (LIM0 mechanism).^7^ Assuming the kinetic parameters of LIM1 implies that the HPALD yield from Crounse *et al*.^6^ is equal to 0.25. In our analysis, the parameter *BF* is used to describe the HPALD/diHPCARP branching fraction of R7a and R7b:
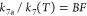
E5

*BF* is equal to the HPALD yield. The fact that HPALD does not recycle OH but diHPCARP does is the reason that E5 is a defined parameter in the system, i.e., the HPALD yield is equal to 1 – OH_yield_. The temperature dependence of *BF* is explored further in the scenarios in the Supporting Information, Section S5.

The MATLAB^34^ program simultaneously analyzed all the OH kinetic traces (94 traces), which were taken over a wide range of temperatures, [isoprene] and [O_2_], to provide a robust test of the LIM1 mechanism. The following parameters have been adjusted to test LIM1: the barriers to OH products (*Ea*_5(2/5)_ and *Ea*_6(3/6)_), the LIM1 R + O_2_ rate coefficients (*k*_3*i*_), scaled using *S*_3,*Z*-δ-RO2_ and *S*_3,other-RO2_, the RO_2_,_*i*=1,6_ binding energy (*E*_–3,adjust_), and *BF*.

In the scenarios given in Section S5 of the Supporting Information, the number of floated parameters is progressively increased until all six parameters are adjusted. In general, all the scenarios provide a good fit to the experimental data based on χ^2^. While these less constrained models do assign defined parameters, the uniqueness of the parameters is debatable as some of the rate coefficients are highly correlated. Sample fits to the traces are shown in Figures 1 and 2, and the results when the six parameters were adjusted (scenario 14) are summarized in Table 1, which includes the LIM1 and Teng *et al.*(4) parameters (see below). The complexity of LIM1 means that product formation occurs on several timescales. However, the essence of product formation can be approximated using *k*(bulk), which we define as equal to ln(2) divided by the time for half the products to form. LIM1 defines it as the product of the weighted equilibrium amount of *Z*-δ peroxy radicals and *k*_6_.^8^ At room temperature, as almost all products are formed *via*R6, both definitions of *k*(bulk) produce similar values.

**Figure 3:**
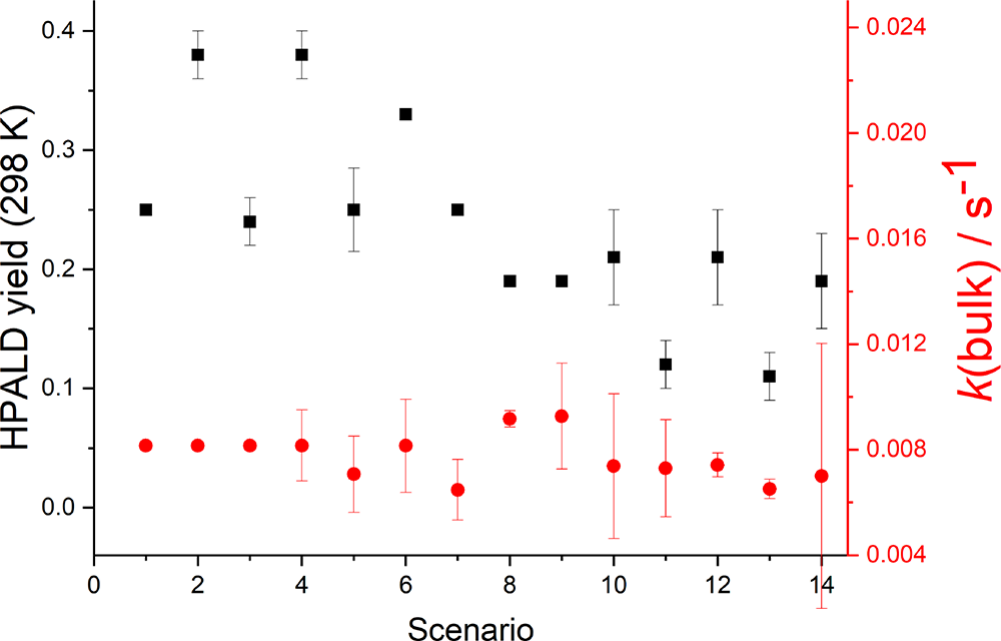
*k*(bulk), defined as ln(2)/the time for half the products to form, and the HPALD yield at 298 K from our results in Table 1, together with the other scenarios given in the Supporting Information. *k*(bulk) is equal to (0.0076 ± 0.0003) s^–1^, almost independent of the scenario. In some scenarios, the HPALD yield was fixed (no error bars) to the literature^4^ (scenarios 1, 6, 7, 8, and 9). While the data are consistent with these fixed HPALD yields, the value of *BF*(298 K) when floated tends to be lower and even lower when *BF* is assigned a temperature dependence (see scenarios 11 and 13). The parameters of scenario 14 are given in Table 1.

The fits to all the traces are given in Section S7 of the Supporting Information. Further analysis is given in the Supporting Information (Section S5) where *S*_3_ and *Ea* are constrained in a number of ways, and the temperature dependence of *BF* is explored. These various scenarios demonstrate that, while there is uncertainty and correlation in the parameters, *k*(bulk) and the HPALD yield (*k*_7a_/*k*_7_) are defined with good confidence (see Figure 3).

## Discussion

**Figure 4:**
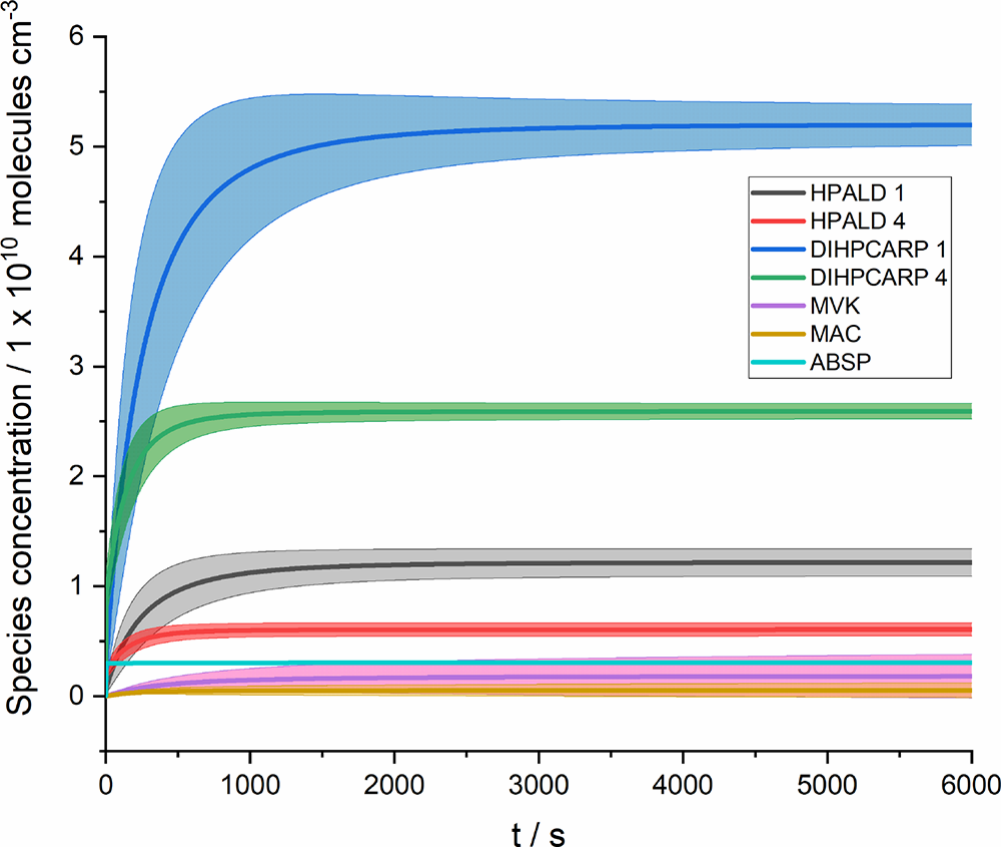
Monte Carlo simulations of the result in Table 1 (scenario 14 in the Supporting Information) at 295 K. [OH]_0_ was equal to 10^11^ molecule cm^–3^ and [isoprene] and [O_2_] were sufficiently large that the result is independent of these concentrations. Any OH product was not allowed to recycle so that, at long times, the sum of all the products is equal to [OH]_0_. ABSP represents the product of a direct hydrogen abstraction from isoprene.

From Table 1, the main feature is that the best-fit barriers to products have been adjusted no more than 4 kJ mol^–1^ from the LIM1 mechanism. The barriers to products sensitively control the kinetics of the system, but adjustments in the barriers can readily be offset by the *k*_3_ scaling parameters, *S*_3_. As noted above, all our models represent a good fit to the data, so there is a question if these extra parameters are unique when the correlation between the parameters is taken into account. A better comparison between the models is the rate coefficient for product formation, *k*_bulk_, which from Figure 3 can be seen to be essentially independent of the model scenarios considered in this study. In fact, scenario 1 is a perfectly good description of the data and this scenario is simply LIM1 with only *S*_3_ (*S*_3,*Z*-δ-RO2_ = *S*_3,other-RO2_) adjusted, where *S*_3_ is equal to 2.9. While *k*_3_ has a significant error (∼40%), its impact on the kinetics is much less sensitive than the barriers to products. The overall effect of the errors in our fitted parameters was investigated by Monte Carlo (MC) simulations, which plotted out the product formation versus time for thousands of simulations. These plots were the result of sampling the parameters based on the correlation matrix determined from the fit to the data. Figure 4 shows the MC result from the parameters given in Table 1, scenario 14, the scenario where the most parameters were floated and hence the maximum uncertainty in the product distribution. These MC simulations were how the half-life and its error were assigned, which in turn were used to calculate *k*(bulk) (see Figure 3).

Also from Table 1, the value of *k*(bulk) from previous studies is given, where it can be seen that this study is in good agreement with LIM1, but not Teng *et al*. The recent study by Novelli *et al.*(27) was not able to explain their OH recycling data using a *k*_bulk_ of 0.002 s^–1^ based on the Master Chemical Mechanism (MCM v3.3.1)^38^ model and subsequently adjusted their model to yield a *k*_bulk_ equal to 0.006 s^–1^, which is in reasonable agreement with this study. These literature values are plotted in the Supporting Information, Figure S6.

**Figure 5:**
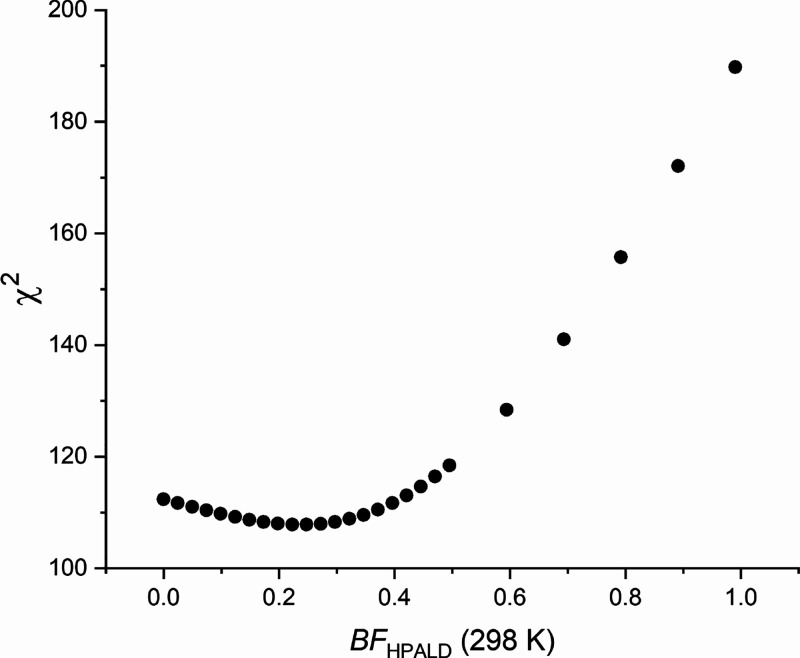
Plot of how well the data is fitted, χ^2^, for a range of fixed branching factors, where only the R + O_2_ scaling factor, *S*_3_, is floated, i.e., scenario 3 with a range of fixed *BF*, using the temperature-dependent *BF*(*T*), S-E3 × *BF*_scaling_. This plot demonstrates that BF at 298 K has a distinct minimum, and poor fits are returned when the *BF* is >0.4.

From Table 1, scenario 14, the branching fraction parameter *BF* (*k*_7a_/*k*_7_) is equal to 0.19. This HPALD yield is in fairly good agreement with that implied by Crounse *et al*.,^4,6^ 0.25, and used in our scenario 1, assuming LIM1. However, Figure 3 shows that the *BF* can take a range of values but always indicates that di-HPCARP is the major product (i.e., *BF<* 0.5). To illustrate how well *BF* is defined, the model (scenario 3) has been run where the *BF* is fixed over the range of 0–1.0 and *S*_3_ is floated (*S*_3,*Z*-δ-RO2_ = *S*_3,other-RO2_), where *BF*(*T*) is taken into account *via* S-E3 × *BF*_scaling_ (see the Supporting Information). Figure 5 shows that χ^2^ has a distinct minimum (ca. 0.25), but between 0.1 and 0.4, the change in χ^2^ is modest. This provides some explanation of why a range of *BF* values can accommodate the data but not *BF* values above 0.4.

Figure 2 illustrates how various mechanisms and scenarios influence a typical decay trace. The orange line in Figure 2 shows the fit to the data if OH from R8 is removed, i.e., the only OH recycling is only *via* the 1,5 H shift. It is a better fit than if there is no recycling at all (black line), but it is still a poor fit. Ultimately, R5 without R8 is not able to fit the data. The value of *BF* in Table 1 assumes that *BF* is independent of temperature. In the Supporting Information, Section S4, reaction rate theory calculations are reported for *Z*,*Z*′-OH-allyl + O_2_ to form HPALD (*k*_7a_) or diHPCARP (*k*_7b_). While these calculations do not identify the absolute rate coefficients, they indicate their relative temperature dependence. Both reaction rate coefficients decrease with increased temperature, but *k*_7b_ shows a slightly greater negative dependence (see Figure S4). As our experiments were conducted at high temperatures (average temperature of ∼509 K), it is likely that the *BF* at 298 K is a little smaller than that given in Table 1. The temperature dependence of the branching fraction, *BF*(*T*), is explored in some of the scenarios (see the Supporting Information, Section S5).

In a recent study by Berndt *et al*.,^26^R1 was studied in a flow tube, where a mass spectrometer was used to detect the products using ion-molecule titration reactions. The products were detected after 7.9 s, which is much less than the half-life of the reaction, ca. 100 s (see Figures 3 and 4). Peroxy radicals, RO_2_, were observed, HPALD was observed to be the major product from RO_2_ isomerization, and its yield was assigned to be 0.76. diHPCARP is expected to decompose (R8) to dihydroperoxide carbonyl (DHP) on their experimental timescale.^8,27^ DHP was observed but only in a small yield, 0.02. Therefore, the results from Berndt *et al*.^26^ are incompatible with our study and that of Crounse *et al.*(6) as they imply that OH traces would exhibit substantially less recycling; HPALD requires a much higher temperature than in our experiments to decompose to OH. The problem with mass spectrometers that use ion-molecule reactions to assign product concentrations is that there is a large uncertainty in the thermochemistry of these reactions; some reactions are endothermic and therefore do not happen, and others are so exothermic that there is essentially 100% fragmentation of the parent ion. Berndt *et al*.’s assigned [RO_2_] was about a factor of 10 below the expected [RO_2_], and their assigned [HPALD] was about half the [RO_2_] when only ∼10% of the reaction has occurred (reaction time is 7.9 s when the half-life is ∼100 s). These problems mean that there are potentially very large uncertainties in the assigned product yields. In the present experiments, OH was directly monitored *via in situ* measurements and the HPALD yield is assigned on the basis that HPALD does not decompose to OH.

Also shown in Figure 2 are fits to the data using the parameter modifications to the LIM1 mechanism in the recent paper by Teng *et al.*(4) and a refinement of this work, the Caltech model.^5^ These models give a significantly worse fit than our best models. This poorer fit is expected as the modifications of Teng *et al.* reduced the importance of the *Z*-δ-OH peroxy radicals, *A*_3,*Z*-δ-RO2_ < *A*_3,other-RO2_, and decreased the RO_2_,_*i*=1,6_ binding energy (*E*_–3,adjust_). These changes reduce the flux *via*R6 and result in a smaller *k*(bulk) (see Table 1 and Figure S6). Teng *et al.*’s study was conducted in an environmental chamber, where the RO_2_ radicals were monitored by adding nitric oxide, NO, to the system:
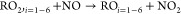
R9a
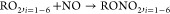
R9b

While R9a is the major channel, R9b produces six nitrates that are linked to the six RO_2_ isomers, which were measured by initially separating them using gas chromatography (GC) and then passing each isomer to a CF_3_O^–^ chemical ionization mass spectrometer (CIMS) for identification. Teng *et al.*’s study was therefore not an *in situ* study, and it was assumed that each of the GC-sampled nitrate isomers was a relative measure of the peroxy radical, RO_2_,_*i*=1,6_, concentrations. However, there is the possibility that the nitrates may interconvert while being GC-separated. Teng *et al.* acknowledged this and also noted that the β-OH nitrates hydrolyzed in the column and corrected their results for these effects. Besides the problem of GC sampling, there is also the possibility that the excess energy from the RO_2_ + NO reaction can lead to isomer scrambling and hence loss of correspondence between relative populations of the nitrates and OH-C_5_H_8_-O_2_ radicals. Teng *et al.* did not consider this RO_2_ + NO isomer scrambling possibility and assumed that all rate coefficients were identical (8.6 × 10^–12^ cm^3^ molecule^–1^ s^–1^), and nitrate yields were also identical, 13%. To fit their data, Teng *et al.* made many parameter adjustments to LIM1, leading to an increased importance of the stable isomers, and their *k*(bulk) is significantly smaller (see Table 1).

Overall, with justifiable and systematic adjustments of the LIM1 parameters, an excellent fit to our data is obtained—see the red line in Figure 2—where OH formed from diHPCARP is the major channel following the 1–6 H shift (R7a and R7b). The parameters in Table 1, together with the scenarios given in the Supporting Information, show that the overall bulk rate coefficient, *k*(bulk), is defined and that the HPALD yield is less than 0.4 (see Figure 5) and more likely equal to the lower values (see Figure 3), which is contrary to the study by Berndt *et al*.^26^

**Figure 6:**
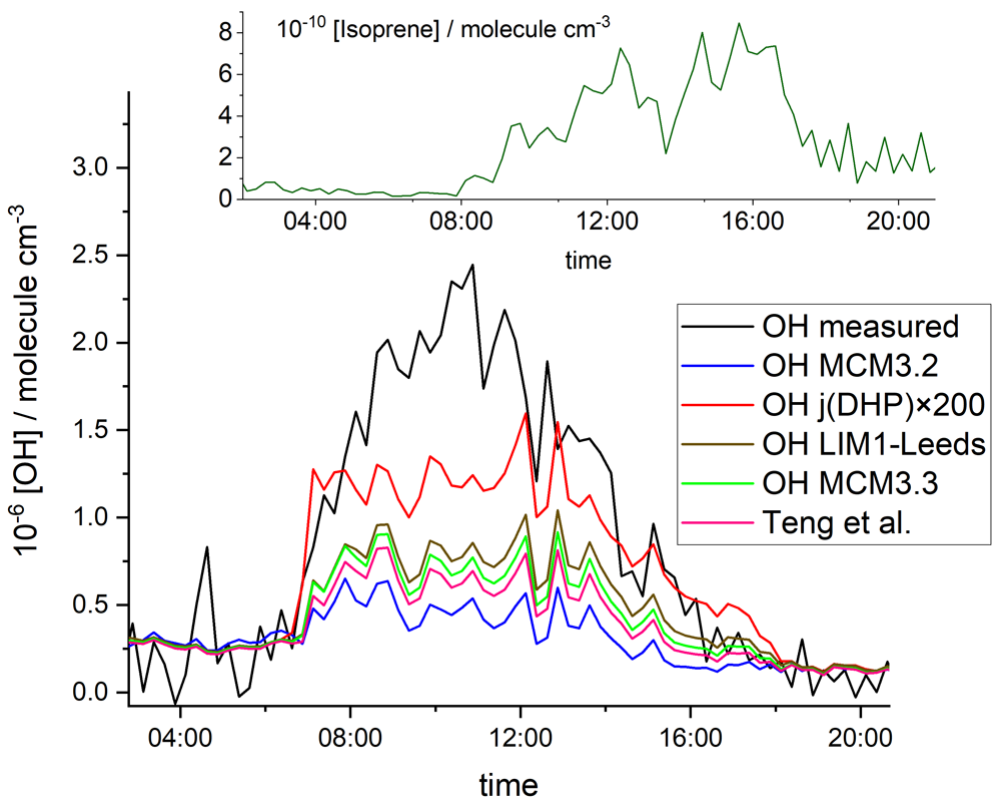
MCM atmospheric model simulations of the OP3 campaign against the actual [OH] measurements (black line). MCM3.2 is the model (blue) before LIM1 and dramatically underestimates the measured [OH]. MCM 3.3.1 (green line) is the model update that includes LIM1. The brown line is the model result that includes the parameters from the current study (LIM1-Leeds), and the pink line is the model using the results of Teng *et al.*(4) The red line is the LIM1-Leeds model with the photolysis of DHP (the products of the diHPCARP decomposition) enhanced using the cross sections in line with those calculated by Liu *et al.*(39) The inset shows the isoprene diurnal profile during the day, where it has not peaked until after 12:00.

With our modifications of the LIM1 parameters, LIM1-Leeds (Table 1), 0-D box modeling (chemistry only) of the OP3 Borneo campaign^28,40^ has been carried out using the MCM description, focusing on OH; the parameters from Teng *et al*.^4^ are also included in the modeling. The results are summarized in Figure 6, where the blue line represents the model that does not incorporate LIM1 (MCM3.2). There is a clear improvement in the [OH] prediction when the LIM1 parameters (MCM3.3) are incorporated (green line), with the present LIM1-Leeds parameters further enhancing the [OH] (maroon line) and the Teng *et al.* parameter reducing the [OH] (pink line). The main reason that the Teng *et al*. model produces less [OH] than MCM3.3 is the slower *k*(bulk) (see Table 1 and Figure S6), and LIM1-Leeds produces more [OH] than MCM3.3 because of the greater yield of diHPCARP.

While there is still a significant gap between LIM1-Leeds and the measured [OH] (black line), the greater importance of the diHPCARP species in LIM1-Leeds provides both chemical and photochemical routes to OH. The co-product of diHPCARP decomposition is dihydroperoxide carbonyl (DHP), which can photolyze to OH, as noted by Peeters *et al.*,^8^ and in the MCM, its photolysis cross sections are assigned to those of a simple peroxide. However, in a recent study by Liu *et al*.,^39^ enhanced photolysis cross sections were observed for the DHP-type molecule, 2-hydroperoxypropanal, where an efficient 1,5 H-shift was identified, resulting in singlet O_2_ and an enol. However, in the case of DHP, the 1,5 H shift can also lead to OH. Figure 6 also includes DHP-enhanced photolysis (orange line), where the photolysis rates have been increased by a factor of 200 above that in the MCM, *j*(DHP) × 200, where it is assumed that each photon produces one OH and a factor of 200 brings the photolysis rates, in line with those reported by Liu *et al.*(39) This enhanced photolysis produces [OH] significantly greater than the other models and is 0.61 of the measured [OH] between 08:00 and 16:00; further increasing the DHP photolysis rate does not increase [OH] (see the Supporting Information, Section S6).

Over the course of the day, the isoprene concentration increases over an order of magnitude—maximum ∼10^11^ molecule cm^3^—so effectively modeling [OH] is becoming more and more an isoprene-only problem, and from Figure 6, it can be seen over the course of the day that the difference between measured and modeled [OH] is progressively decreasing. Therefore, early in the day, the enhanced [OH] is more likely linked to the photolysis of an OH precursor that has accumulated overnight.

Overall, the chemistry and photochemistry of DHP (and its subsequent products) are uncertain, so there is scope to enhance [OH]. Most other channels in isoprene oxidation chemistry are sufficiently well known such that they do not have the scope of reconciling the measured and modeled OH, which from Figure 6 can be seen to converge over the day as the concentration of isoprene increases. However, it has been suggested that peroxy2 (see Figure 1) is formed with so much energy that it can decompose to OH and hydroxyperoxy carbonyl epoxide, e.g., Berndt *et al.*(26) This alternative OH source will have no impact on the present work as it arises from the same channel of reaction 7 that produces DHP and OH but might affect the results of Figure 6 depending on the relative cross sections of DHP and the hydroxyperoxy carbonyl epoxide.

## Conclusions

Time-resolved experiments have been carried out that have monitored OH in the presence of isoprene and oxygen at elevated temperatures, 420–584 K. Under these conditions, distinct OH recycling was directly observed on the millisecond timescale. These experiments should be free from sampling artifacts and secondary radical–radical chemistry. The observed OH recycling is in agreement with the theory-based Leuven Isoprene Mechanism (LIM1), and data analysis of the OH traces demonstrated that only small adjustments of the LIM1 rate coefficients were required to fit our data. Our refined parameters, LIM1-Leeds, predict at 298 K that the timescale for product formation is essentially the same as LIM1 and in reasonable agreement with Novelli *et al.*(27) but is about four times faster than the recent study by Teng *et al.*(4) In addition, this study predicts that diHPCARP, and not HPALD, is the major product of reaction, which is contrary to the recent study by Berndt *et al.*(26) Our results have been inputted into an atmospheric chemistry model and further improve the agreement between modeled and measured [OH], especially as the conditions better approximate to an isoprene-only system.

## Supporting Information Available

LIM1 mechanism for addition to the unsubstituted double bond in isoprene, the complete mechanism used in MATLAB fitting, experimental details, master equation modeling to demonstrate pressure independence, details on data analysis models (scenarios), further MCM modeling, and a summary of experimental data (PDF)

## References

[ref1] Goldstein A. H.; Galbally I. E. Known and Unexplored Organic Constituents in the Earth’s Atmosphere. Environ. Sci. Technol. 2007, 41, 1514–1521. 10.1021/es072476p. 17396635

[ref2] Guenther A.; Karl T.; Harley P.; Wiedinmyer C.; Palmer P. I.; Geron C. Estimates of global terrestrial isoprene emissions using MEGAN (Model of Emissions of Gases and Aerosols from Nature). Atmos. Chem. Phys. 2006, 6, 3181–3210. 10.5194/acp-6-3181-2006.

[ref3] Medeiros D. J.; Blitz M. A.; James L.; Speak T. H.; Seakins P. W. Kinetics of the reaction of OH with isoprene over a wide range of temperature and pressure including direct observation of equilibrium with the OH adducts. J. Phys. Chem. A 2018, 122, 7239–7255. 10.1021/acs.jpca.8b04829. 30137992

[ref4] Teng A. P.; Crounse J. D.; Wennberg P. O. Isoprene Peroxy Radical Dynamics. J. Am. Chem. Soc. 2017, 139, 5367–5377. 10.1021/jacs.6b12838. 28398047

[ref5] Wennberg P. O.; Bates K. H.; Crounse J. D.; Dodson L. G.; McVay R. C.; Mertens L. A.; Nguyen T. B.; Praske E.; Schwantes R. H.; Smarte M. D.; St Clair J. M.; Teng A. P.; Zhang X.; Seinfeld J. H. Gas-Phase Reactions of Isoprene and Its Major Oxidation Products. Chem. Rev. 2018, 118, 3337–3390. 10.1021/acs.chemrev.7b00439. 29522327

[ref6] Crounse J. D.; Paulot F.; Kjaergaard H. G.; Wennberg P. O. Peroxy radical isomerization in the oxidation of isoprene. Phys. Chem. Chem. Phys. 2011, 13, 13607–13613. 10.1039/c1cp21330j. 21701740

[ref7] Peeters J.; Nguyen T. L.; Vereecken L. HOx radical regeneration in the oxidation of isoprene. Phys. Chem. Chem. Phys. 2009, 11, 5935–5939. 10.1039/b908511d. 19588016

[ref8] Peeters J.; Müller J. F.; Stavrakou T.; Nguyen V. S. Hydroxyl Radical Recycling in Isoprene Oxidation Driven by Hydrogen Bonding and Hydrogen Tunneling: The Upgraded LIM1 Mechanism. J. Phys. Chem. A 2014, 118, 8625–8643. 10.1021/jp5033146. 25010574

[ref9] Paulot F.; Crounse J. D.; Kjaergaard H. G.; Kürten A.; St. Clair J. M.; Seinfeld J. H.; Wennberg P. O. Unexpected Epoxide Formation in the Gas-Phase Photooxidation of Isoprene. Science 2009, 325, 730–733. 10.1126/science.1172910. 19661425

[ref10] Carlton A. G.; Wiedinmyer C.; Kroll J. H. A review of Secondary Organic Aerosol (SOA) formation from isoprene. Atmos. Chem. Phys. 2009, 9, 4987–5005. 10.5194/acp-9-4987-2009.

[ref11] Heard D. E. Atmospheric field measurements of the hydroxyl radical using laser-induced fluorescence spectroscopy. Annu. Rev. Phys. Chem. 2006, 57, 191–216. 10.1146/annurev.physchem.57.032905.104516. 16599809

[ref12] Finlayson-Pitts B. J.; Pitts J. N., Chemistry of the upper and lower atmosphere : theory, experiments, and applications; Academic Press: San Diego, California, USA. London, UK, 2000.

[ref13] Jones C. E.; Hopkins J. R.; Lewis A. C. In situ measurements of isoprene and monoterpenes within a south-east Asian tropical rainforest. Atmos. Chem. Phys. 2011, 11, 6971–6984. 10.5194/acp-11-6971-2011.

[ref14] Lelieveld J.; Butler T. M.; Crowley J. N.; Dillon T. J.; Fischer H.; Ganzeveld L.; Harder H.; Lawrence M. G.; Martinez M.; Taraborrelli D.; Williams J. Atmospheric oxidation capacity sustained by a tropical forest. Nature 2008, 452, 737–740. 10.1038/nature06870. 18401407

[ref15] Kubistin D.; Harder H.; Martinez M.; Rudolf M.; Sander R.; Bozem H.; Eerdekens G.; Fischer H.; Gurk C.; Klüpfel T.; Königstedt R.; Parchatka U.; Schiller C. L.; Stickler A.; Taraborrelli D.; Williams J.; Lelieveld J. Hydroxyl radicals in the tropical troposphere over the Suriname rainforest: comparison of measurements with the box model MECCA. Atmos. Chem. Phys. 2010, 10, 9705–9728. 10.5194/acp-10-9705-2010.

[ref16] Sanchez D.; Jeong D.; Seco R.; Wrangham I.; Park J. H.; Brune W. H.; Koss A.; Gilman J.; de Gouw J.; Misztal P.; Goldstein A.; Baumann K.; Wennberg P. O.; Keutsch F. N.; Guenther A.; Kim S. Intercomparison of OH and OH reactivity measurements in a high isoprene and low NO environment during the Southern Oxidant and Aerosol Study (SOAS). Atmos. Environ. 2018, 174, 227–236. 10.1016/j.atmosenv.2017.10.056.

[ref17] Tan Z. F.; Fuchs H.; Lu K. D.; Hofzumahaus A.; Bohn B.; Broch S.; Dong H. B.; Gomm S.; Haseler R.; He L. Y.; Holland F.; Li X.; Liu Y.; Lu S. H.; Rohrer F.; Shao M.; Wang B. L.; Wang M.; Wu Y. S.; Zeng L. M.; Zhang Y. S.; Wahner A.; Zhang Y. H. Radical chemistry at a rural site (Wangdu) in the North China Plain: observation and model calculations of OH, HO_2_ and RO_2_ radicals. Atmos. Chem. Phys. 2017, 17, 663–690. 10.5194/acp-17-663-2017.

[ref18] Whalley L. K.; Edwards P. M.; Furneaux K. L.; Goddard A.; Ingham T.; Evans M. J.; Stone D.; Hopkins J. R.; Jones C. E.; Karunaharan A.; Lee J. D.; Lewis A. C.; Monks P. S.; Moller S. J.; Heard D. E. Quantifying the magnitude of a missing hydroxyl radical source in a tropical rainforest. Atmos. Chem. Phys. 2011, 11, 7223–7233. 10.5194/acp-11-7223-2011.

[ref19] Fuchs H.; Hofzumahaus A.; Rohrer F.; Bohn B.; Brauers T.; Dorn H. P.; Haseler R.; Holland F.; Kaminski M.; Li X.; Lu K.; Nehr S.; Tillmann R.; Wegener R.; Wahner A. Experimental evidence for efficient hydroxyl radical regeneration in isoprene oxidation. Nat. Geosci. 2013, 6, 1023–1026. 10.1038/ngeo1964.

[ref20] Winiberg F. A. F.; Dillon T. J.; Orr S. C.; Gross C. B. M.; Bejan I.; Brumby C. A.; Evans M. J.; Smith S. C.; Heard D. E.; Seakins P. W. Direct measurements of OH and other product yields from the HO_2_ + CH_3_C(O)O_2_ reaction. Atmos. Chem. Phys. 2016, 16, 4023–4042. 10.5194/acp-16-4023-2016.

[ref21] Da Silva G.; Graham C.; Wang Z. F. Unimolecular beta-Hydroxyperoxy Radical Decomposition with OH Recycling in the Photochemical Oxidation of Isoprene. Environ. Sci. Technol. 2010, 44, 250–256. 10.1021/es900924d. 19943615

[ref22] Mutzel A.; Poulain L.; Berndt T.; Iinuma Y.; Rodigast M.; Boge O.; Richters S.; Spindler G.; Sipila M.; Jokinen T.; Kulmala M.; Herrmann H. Highly Oxidized Multifunctional Organic Compounds Observed in Tropospheric Particles: A Field and Laboratory Study. Environ. Sci. Technol. 2015, 49, 7754–7761. 10.1021/acs.est.5b00885. 26011767

[ref23] Schervish M.; Donahue N. M. Peroxy radical chemistry and the volatility basis set. Atmos. Chem. Phys. 2020, 20, 1183–1199. 10.5194/acp-20-1183-2020.

[ref24] Liu Z.; Nguyen V. S.; Harvey J.; Muller J. F.; Peeters J. Theoretically derived mechanisms of HPALD photolysis in isoprene oxidation. Phys. Chem. Chem. Phys. 2017, 19, 9096–9106. 10.1039/C7CP00288B. 28317054

[ref25] Wolfe G. M.; Crounse J. D.; Parrish J. D.; St. Clair J. M.; Beaver M. R.; Paulot F.; Yoon T. P.; Wennberg P. O.; Keutsch F. N. Photolysis, OH reactivity and ozone reactivity of a proxy for isoprene-derived hydroperoxyenals (HPALDs). Phys. Chem. Chem. Phys. 2012, 14, 7276–7286. 10.1039/c2cp40388a. 22517221

[ref26] Berndt T.; Hyttinen N.; Herrmann H.; Hansel A. First oxidation products from the reaction of hydroxyl radicals with isoprene for pristine environmental conditions. Commun. Chem. 2019, 2, 21 10.1038/s42004-019-0120-9.

[ref27] Novelli A.; Vereecken L.; Bohn B.; Dorn H. P.; Gkatzelis G. I.; Hofzumahaus A.; Holland F.; Reimer D.; Rohrer F.; Rosanka S.; Taraborrelli D.; Tillmann R.; Wegener R.; Yu Z. J.; Kiendler-Scharr A.; Wahner A.; Fuchs H. Importance of isomerization reactions for OH radical regeneration from the photo-oxidation of isoprene investigated in the atmospheric simulation chamber SAPHIR. Atmos. Chem. Phys. 2020, 20, 3333–3355. 10.5194/acp-20-3333-2020.

[ref28] Stone D.; Evans M. J.; Edwards P. M.; Commane R.; Ingham T.; Rickard A. R.; Brookes D. M.; Hopkins J.; Leigh R. J.; Lewis A. C.; Monks P. S.; Oram D.; Reeves C. E.; Stewart D.; Heard D. E. Isoprene oxidation mechanisms: measurements and modelling of OH and HO_2_ over a South-East Asian tropical rainforest during the OP3 field campaign. Atmos. Chem. Phys. 2011, 11, 6749–6771. 10.5194/acp-11-6749-2011.

[ref29] Onel L.; Blitz M. A.; Seakins P. W. Direct Determination of the Rate Coefficient for the Reaction of OH Radicals with Monoethanol Amine (MEA) from 296 to 510 K. J. Phys. Chem. Lett. 2012, 3, 853–856. 10.1021/jz300200c. 26286410

[ref30] Glowacki D. R.; Lockhart J.; Blitz M. A.; Klippenstein S. J.; Pilling M. J.; Robertson S. H.; Seakins P. W. Interception of Excited Vibrational Quantum States by O_2_ in Atmospheric Association Reactions. Science 2012, 337, 1066–1069. 10.1126/science.1224106. 22936771

[ref31] Speak T. H.; Blitz M. A.; Stone D.; Seakins P. W. A new instrument for time-resolved measurement of HO_2_ radicals. Atmos. Meas. Tech. 2020, 13, 839–852. 10.5194/amt-13-839-2020.

[ref32] Stone D.; Blitz M.; Ingham T.; Onel L.; Medeiros D. J.; Seakins P. W. An instrument to measure fast gas phase radical kinetics at high temperatures and pressures. Rev. Sci. Instrum. 2016, 87, 054102. 27250442 10.1063/1.4950906

[ref33] Medeiros D. J.; Robertson S. H.; Blitz M. A.; Seakins P. W. Direct Trace Fitting of Experimental Data Using the Master Equation: Testing Theory and Experiments on the OH + C_2_H_4_ Reaction. J. Phys. Chem. A 2020, 124, 4015–4024. 10.1021/acs.jpca.0c02132. 32353235

[ref34] MATLAB and Optimization Toolbox Release R2016a. Natwick: Math Works Inc., 2016.

[ref35] Beechem J. M.; Knutson J. R.; Brand L. Global Analysis of Multiple Dye Fluorescence Anisotropy Experiments on Proteins. Biochem. Soc. Trans. 1986, 14, 832–835. 10.1042/bst0140832. 3781080

[ref36] Shampine L. F.; Reichelt M. W. The MATLAB ODE Suite. SIAM J. Sci. Comput. 1997, 18, 1–22. 10.1137/S1064827594276424.

[ref37] Moré J. J.; Sorensen D. C. Computing a Trust Region Step. SIAM J. Sci. Stat. Comput. 1983, 4, 553–572. 10.1137/0904038.

[ref38] Jenkin M. E.; Young J. C.; Rickard A. R. The MCM v3.3.1 degradation scheme for isoprene. Atmos. Chem. Phys. 2015, 15, 11433–11459. 10.5194/acp-15-11433-2015.

[ref39] Liu Z.; Nguyen V. S.; Harvey J.; Muller J. F.; Peeters J. The photolysis of alpha-hydroperoxycarbonyls. Phys. Chem. Chem. Phys. 2018, 20, 6970–6979. 10.1039/C7CP08421H. 29465129

[ref40] Edwards P. M.; Evans M. J.; Furneaux K. L.; Hopkins J.; Ingham T.; Jones C.; Lee J. D.; Lewis A. C.; Moller S. J.; Stone D.; Whalley L. K.; Heard D. E. OH reactivity in a South East Asian tropical rainforest during the Oxidant and Particle Photochemical Processes (OP3) project. Atmos. Chem. Phys. 2013, 13, 9497–9514. 10.5194/acp-13-9497-2013.

