# Supplementary material for: Direct Measurements of Isoprene Autoxidation: Pinpointing Atmospheric Oxidation in Tropical Forests
Source: JACS Au. 2022 Mar 18;2(4):809–18. doi: 10.1021/jacsau.1c00525 (PMC9088302; doi:10.1021/jacsau.1c00525)
Supplement: Supplementary file 1 — au1c00525_si_001.pdf [file au1c00525_si_001.pdf]

Direct measurements of isoprene autoxidation: pinpointing atmospheric oxidation in tropical forests

Diogo J Medeiros[1], Mark A Blitz*[1,2], Paul W Seakins[1] and Lisa K Whalley[1,2]

1 - School of Chemistry, University of Leeds, Leeds, LS2 9JT, UK

2 - National Centre for Atmospheric Science (NCAS), University of Leeds, Leeds, LS2 9JT, UK

Supporting Information

Contents

1. LIM1 mechanism for addition to the unsubstituted double bond in isoprene
2. Complete mechanism used in MATLAB fitting.
3. Experimental details
4. Master equation modelling
5. Details on data analysis models (scenarios)
6. Further MCM modelling
7. Summary of experimental data
8. References for Supplementary material

1. Mechanism from LIM1 for Addition of OH to the non-substituted double bond.

Scheme SI1 shows the LIM1 mechanism [1] for addition of the OH to the non-methyl end of isoprene; the other OH addition is given in the main text, Figure 1.

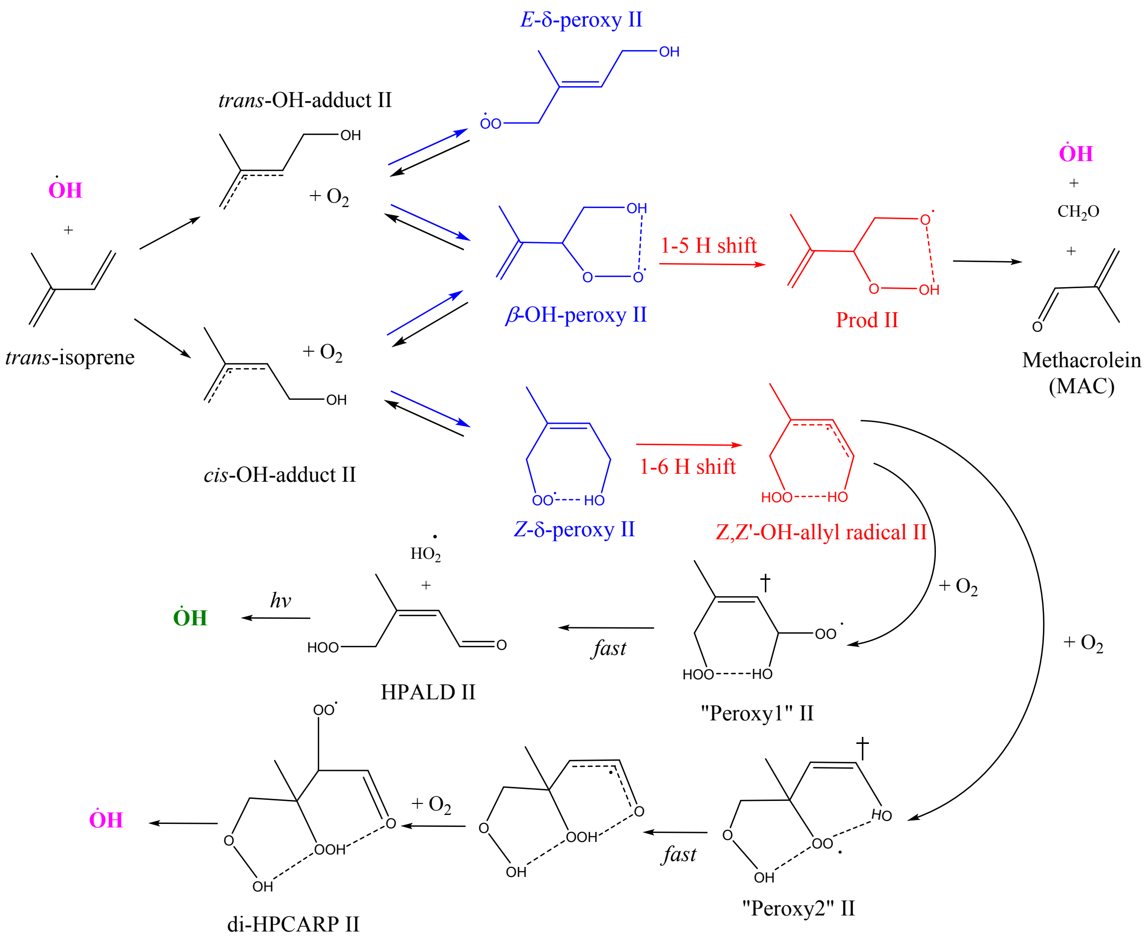

Scheme SI1. The Leuven Isoprene Mechanism 1 as proposed by Peeters *et al.*[1] Case II – OH addition to carbon 4 of the primary chain.

2. Complete reaction mechanism used for MATLAB data fitting

All 32 rate coefficients of the LIM1 were described using a generic four-parameter format:

$$k_i = A_i \times (T/298\ \mathrm{K})^{n_i} \times \exp\left(\frac{Ea_i}{RT}\right) \times \exp\left(\frac{Q_i}{T^3}\right) \qquad \text{SI-E1}$$

This format is flexible enough to generate all the required rate coefficients, e.g. setting n_i, Ea_i or Q_i (the specific tunneling contribution, which is discussed further in section 4) to zero reduces the equation to a simpler form. The LIM1 mechanism and its rate coefficients are

explicitly described using the following schemes where we have used the LIM1 nomenclature, and for ease of reading, have separated OH addition at the substituted double bond (Figure 1, CASE I), OH additional the non-substituted double bond (Figure SI1, CASE II) and experimental parameters :

Table S1 – Reaction mechanism used to fit experimental data. Nomenclature follows that of the LIM1 mechanism, where adjusted parameters are colour highlighted.

Case I – addition of the OH to the substituted double bond

Reaction in LIM1	Equation (variable parameters shaded)	Rate coefficient at 298 K from LIM1
OH + isoprene ⟶ *trans*-OH-adduct I	$k_{1,C1a}$ = 0.355*9.5E-11(*T*/298)^-1.33	
OH + isoprene ⟶ *cis*-OH-adduct I	$k_{1,C1b}$ = 0.355*9.5E-11(*T*/298)^-1.33	
trans-OH-adduct I + O_2 ⟶ *E*-δ-OH-peroxy I	$k_{3\text{-}E\text{-}\delta}$ = S_{3other}*0.5E-12*EXP(-480/*T*)	1.0e-13
trans-OH-adduct I + O_2 ⟶ β-OH-peroxy I	$k_{3\text{-}\beta\text{-}A}$ = S_{3other} *0.6E-12	6.0e-13
E-δ-OH-peroxy I ⟶ *trans*-OH-adduct I + O_2	$k_{\text{-}3\text{-}E\text{-}\delta}$ = 5.714E13*EXP(-9028/*T*)	4.0
β-OH-peroxy I ⟶ OH + MVK	$k_{5,I}$ = 1.038E11*EXP(-(9746.4 + Ea_5)/*T*)	6.5e-4
β-OH-peroxy I ⟶ *trans*-OH-adduct I + O_2	$k_{\text{-}3\text{-}\beta\text{-}A}$ = 1.71E15*EXP(-10743/*T*)	0.38
β-OH-peroxy I ⟶ *cis*-OH-adduct I + O_2	$k_{\text{-}3\text{-}\beta\text{-}B}$ = 1.724E15*EXP(-11322/*T*)	0.054
cis-OH-adduct I + O_2 ⟶ β-OH-peroxy I	$k_{3\text{-}\beta\text{-}B}$ = S_{3other} *0.6E-12	6.0e-13
cis-OH-adduct I + O_2 ⟶ Z-δ-OH-peroxy I	$k_{3\text{-}Z\text{-}\delta}$ = $S_{3\text{-}Z\text{-}\delta}$ *0.7E-12	7.0e-13
Z-δ-OH-peroxy I ⟶ Z,Z-O-allyl I	$k_{6,I}$ = 3.556E10*EXP(-(8590.5 + Ea_6)/*T*)* EXP(1.0265E8/*T*^3)	0.52
Z-δ-OH-peroxy I ⟶ *cis*-OH-adduct I + O_2	$k_{\text{-}3\text{-}Z\text{-}\delta}$ = 1.043E15*EXP(-9837.6/*T*)	4.8
Z,Z-O-allyl I + O_2 ⟶ peroxy2 I	k_{7b} = 4e-14*(1-*BF*)	
Z,Z-O-allyl I + O_2 ⟶ HPALD + HO_2	k_{7a} = 4e-14**BF*	
Peroxy2 I + O_2 ⟶ di-HPCARP I	k_8 = >4e-14	
di-HPCARP I ⟶ OH + products	k_9 = 5E13*EXP(-10069.8/T)	

Case II – addition of the OH to the unsubstituted double bond

Reaction in LIM1	Equation (variable parameters shaded)	Rate coefficient at 298 K from LIM1
OH + isoprene → *trans*-OH-adduct II	$k_{1,C1a}$ = 0.097*9.5E-11(T/298)^-1.33	
OH + isoprene → *cis*-OH-adduct II	$k_{1,C1b}$ = 0.233*9.5E-11(T/298)^-1.33	
trans-OH-adduct II + O_2 → *E*-δ-OH-peroxy II	$k_{3\text{-E-}\delta}$ = S_{3other} *0.5E-12*EXP(-480/T)	1.0e-13
trans-OH-adduct II + O_2 → β-OH-peroxy II	$k_{3\text{-}\beta\text{-A}}$ = S_{3other}*0.7E-12	7.0e-13
E-δ-OH-peroxy II → *trans*-OH-adduct II + O_2	$k_{-3\text{-E-}\delta}$ = 4.25E13*EXP(-9984/T)	0.12
β-OH-peroxy II → OH + MACR	$k_{5,II}$ = 1.877E11*EXP(-(9751.7+ Ea_5)/T)	6.5e-4
β-OH-peroxy II → *trans*-OH-adduct II + O_2	$k_{-3\text{-}\beta\text{-A}}$ = 2.1E15*EXP(-11569/T)	0.026
β-OH-peroxy II → *cis*-OH-adduct II + O_2	$k_{-3\text{-}\beta\text{-B}}$ = 2.10E15*EXP(-11705/T)	0.018
cis-OH-adduct II + O_2 → β-OH-peroxy II	$k_{3\text{-}\beta\text{-B}}$ = S_{3other} *0.7E-12	7.0e-13
cis-OH-adduct II + O_2 → Z-δ-OH-peroxy II	$k_{3\text{-Z-}\delta}$ = $S_{3\text{-Z-}\delta}$ *0.4E-12	4.0e-13
Z-δ-OH-peroxy II → Z,Z-O-allyl II	$k_{6,II}$ = 1.07E11*EXP(-(8174.3 + Ea_6)/T)* EXP(1.03E8/T^3)	5.7
Z-δ-OH-peroxy II → *cis*-OH-adduct II + O_2	$k_{-3\text{-Z-}\delta}$ = 6.12E14*EXP(-10254/T)	0.70
Z,Z-O-allyl II + O_2 → peroxy2 I	k_{7b} = 4e-14*(1-BF)	
Z,Z-O-allyl II + O_2 → HPALD + HO_2	k_{7a} = 4e-14*BF	
Peroxy2 II + O_2 → di-HPCARP II	k_8 = >4e-14	
di-HPCARP II → OH + products	k_9 = 5E13*EXP(-10069.8/T)	

Additional Experimental Parameters

Reaction	Equation
OH Diffusional loss	Variable with conditions
OH abstraction from isoprene	1.3E-11*EXP(-434.2/T) [2]

3. Experimental Details

Experiments were carried out in two distinctly different apparatus, where the main difference is how the hydroxyl radical, OH, is measured. In the conventional, low-pressure apparatus,[3,4] the OH is directly detected *in situ* in the reaction zone defined by the overlap of the, photolysis laser and the probe laser, see Figure SI1. In the high pressure apparatus,[5] the photolysis laser creates OH along the length of the reactor, but the OH is not measured *in situ*. The OH is sampled at the end of the reactor by passing through a pinhole into a low-pressure cell, where it is detected by laser induced fluorescence, LIF, see Figure SI2. This technique is known as fluorescence assay by gas expansion, FAGE, and is well known in the field measurement community.[6] The main difference in the present apparatus is that the OH is detected within 1 cm of the pinhole, where the gas is jetting, i.e. undergoing relatively few collisions. We have demonstrated that in this configuration the OH is sampled in ~20 microseconds and the kinetics are essentially unperturbed, i.e. identical to the kinetics in the low-pressure *in situ* OH cell.[2,4,5] In the high-pressure apparatus, the reaction zone is typically the volume within 1 – 2 mm from the pinhole as the experiment is complete is 10 milliseconds.

The reason for deploying these two types of apparatus is because large [O_2] have to be added to the system, see Table S1, to shift the R + O_2 equilibrium towards peroxy radical formation. In the low pressure apparatus, the O_2 quenches the OH fluorescence signal to such an extent that the measured OH is poor quality above [O_2] > 10^{18} molecule cm^{-3}. As the high-pressure apparatus uses the FAGE technique, the effective [O_2] where OH is measured is orders of magnitude lower compared to the low-pressure experiment. Therefore, as the [O_2] is increased there is an amount where the OH signal is better in the high-pressure apparatus, hence the use of this apparatus.

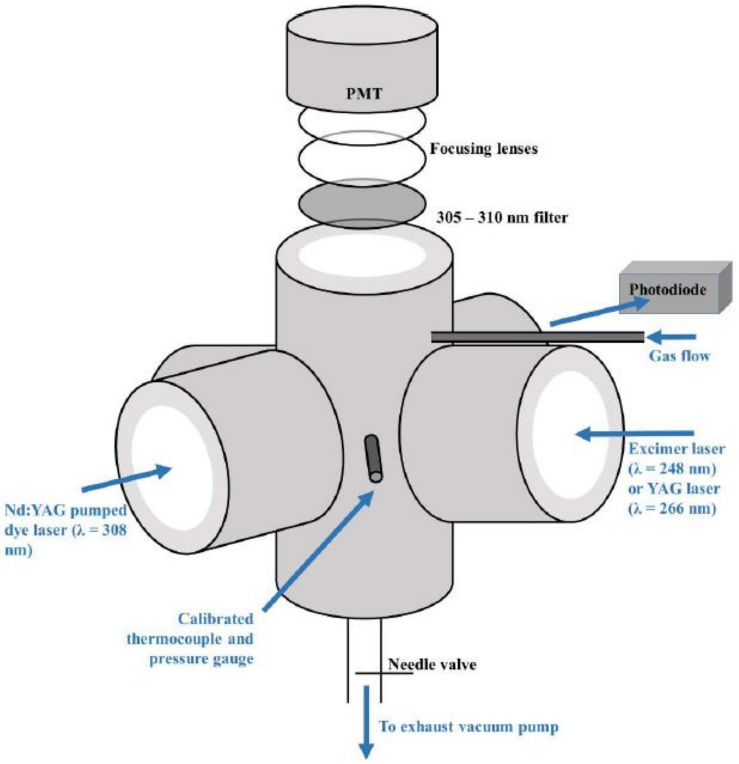

Figure SI1. Schematic of low-pressure reactor: the laser beams cross in the centre of the heated, multi-axes cell and defines the reaction zone where the OH is formed and monitored *in situ*, via detecting the fluorescence at right angles to the laser beams.

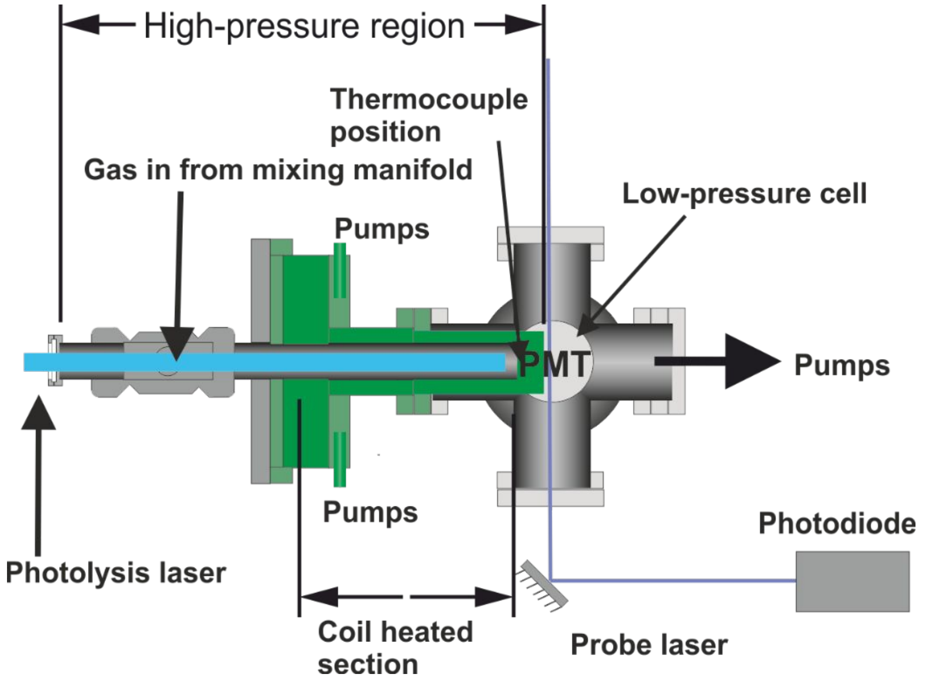

Figure SI2. Schematic of high-pressure reactor.[5] The photolysis laser illuminates the heated reactor (green), where the reaction zone is defined by the volume within ~1-2 mm of the pinhole. The OH is detected in the low-pressure cell, blue, within 1-2 cm of the pinhole where the probed gas is jetting.

Besides where the OH is measured, both experimental apparatus operate on similar principles. Known amounts of gases (H_2O_2, isoprene, O_2 and N_2) are flowed into the reactor using calibrated mass flow controllers, where the gases reach a pre-set temperature controlled by the heaters of the reactor. The reaction is initiated by a photolysis laser, which is either the 248 nm output from a KrF excimer laser (Lambda Physik, LPX200) or the 266 nm output from a Nd:YAG laser (Quantel, Q-smart 850). The photolysis laser dissociates hydrogen peroxide and is a clean instant OH source:

$$H_2O_2 \xrightarrow{\text{248 or 266 nm}} OH + OH \qquad \text{S-R1}$$

The OH is probed (typically at ~308 nm) using a tuneable wavelength dye laser (Sirah, PrecisionScan) by laser induced fluorescence (LIF); the resulting resonant fluorescence is passed through an optical filter (308 ± 5 nm, Barr Associates) and is detected via a photomultiplier (Electron Tubes) situated at right-angles to the plane of the lasers. Additionally, some measurements were made via off resonant LIF, probing at 282 nm to generate the vibrationally excited A state and again monitoring at ~308 nm. In either case, the OH signal is a relative measure of the OH concentration and its time profile is determined by scanning the delay between the firing of the photolysis and dye lasers using a delay generator (BNC 555). The OH trace is the resultant of typically 200 time points, averaging between 3 -12 at each point (see Figure 2 for example traces).

Further details on the low-pressure cell can be found in a number of publications [3, 4] All the low pressure experiments were carried out between 100 – 200 Torr total pressure. The high-pressure apparatus is a more recent development but it has been described in detail in two recent publications.[5, 7] The reactor consists of 2.0 cm diameter stainless tube, lined with a quartz tube. There is a quartz window at one end to admit the photolysis light and the other end is open and is close to a pinhole. The majority of the gas, at ~2 atmospheres, is pumped away but a representative sample flows through the pinhole into the low pressure cell, which is pumped with a roots blower down to a pressure below 1 Torr. The gas emerging through

the pinhole supersonically expands and a cold jet is established for 1 - 2 cm; dependent on pressure drop. It is in this jet where the OH is detected by LIF. As noted above, the OH measured in this zone is wholly representative of the kinetics in the high pressure part of the reaction, and while there is a sampling time it has been shown that reliable kinetics are obtained for processes with half-lives as short as 20 microseconds.[5]

For both systems, H_2O_2 is delivered by a pressurized bubbler situated before a mass flow controller, MFC. A significant amount of the H_2O_2 is lost passing through the narrow metal constrictions of the MFC. The $[H_2O_2]$ in the reactor can readily be determined by monitoring the OH in the absence of isoprene:

$$OH + H_2O_2 \rightarrow H_2O + HO_2 \qquad \text{S-R2}$$

The OH removal kinetics for S-R2 follow a simple signal exponential loss:

$$[OH]_t = [OH]_0 e^{-k_{loss}t} \qquad \text{S-E2}$$

where k_{loss} is the pseudo-first order removal rate coefficient. It is equal to $k_{S\text{-}R2}[H_2O_2]$, and as $k_{S\text{-}R2}$ is well known [8] ($k_{298\,K}$ = 1.7×10^{-12} cm^3 molecule^{-1} s^{-1}, $k(T)$ = 2.9 × 10^{-12}exp(160/T)^{IUPAC}) so $[H_2O_2]$ can be assigned. The values of k_{loss} for each T and p are given in Table SI1; note the k_{loss} is less in the low-pressure apparatus as OH is detected with greater sensitivity when no O_2 is added.

O_2 (99.9%) and N_2 (OFN) were used directly from the cylinder. Isoprene (Sigma-Aldrich) was degassed via freeze-pump-thaw cycling and the diluted (N_2) in either a glass bulb or cylinder. 50% H_2O_2 (Sigma-Aldrich) was bubbled through a mass flow controller before entering the reactors, but required some time (days) before it delivered sufficient $[H_2O_2]$ to the reactor.

Table SI1 Experimental conditions for the 94 OH kinetic traces measured in the presence of isoprene and O_2. In general, the $[H_2O_2]$ in the low-pressure experiments is typically 10^{14} molecule cm^{-3} and the excimer laser brings about ≤ 0.01 photolysis. This implies [OH] ~ 10^{12} molecule cm^{-3}.

T / K	p / Torr	k_{loss} / s^{-1}	$[O_2]$ [(a)]	[Isoprene][(a)]	n_{traces}
420	1404	1362	7.68×10^{18}	$1.33 - 5.42 \times 10^{14}$	4
477	1404	1480	5.85×10^{18}	$1.02 - 8.10 \times 10^{14}$	8
478	134	396	$4.3 – 11.7 \times 10^{17}$	3.4×10^{14}	5
480	125	234	$0.17 – 11 \times 10^{17}$	$1.64 – 5.38 \times 10^{14}$	21
498	1404	1458	5.55×10^{18}	$0.975 – 5.1 \times 10^{14}$	8
513	162	31	9.3×10^{17}	$2.1 – 5.6 \times 10^{14}$	8
543	140	334	$0.27 - 13 \times 10^{17}$	1.44×10^{14}	10
543	140	335	$0.83 - 13 \times 10^{17}$	$0.73 – 2.9 \times 10^{14}$	12
550	1366	1693	4.95×10^{18}	$0.674 - 7.76 \times 10^{14}$	8
584	124	89	9.3×10^{17}	$0.24 - 0.81 \times 10^{14}$	10

[(a)] Units = molecule cm^{-3}

4. Master equation modelling

a) Overall lack of pressure dependence and tunnelling

Although the experiments have been carried out at low and high pressure, it has been assumed in the data analysis that the kinetics of LIM1 are independent of total pressure. This assumption has been tested by performing some master equation simulations of the 1-5 and 1-6 H shift reactions, R5 and R6, starting from OH-isoprene + O_2 using the MESMER [9] software package.

The loss of OH-isoprene from the system is made up of several rate coefficients: RO_2 formation, re-dissociation and reaction to products. All these individual rate coefficients are

pressure dependent, but the overall rate of formation of products is complex (a mixture of the rate coefficients given by the eigenvalue of the system) and is pressure independent. This result is true over all temperatures (250 – 600 K) and pressures (50 – 10^6 Torr) relevant to the present study, see Table SI1. This eigenvalue is equal to a sum of the equilibrium amount of R that directly “well-skips”[10] to products plus the equilibrium amount of RO_2 that thermally reacts to products. The contribution from each of these terms varies between low and high pressure, but the sum is constant, and hence pressure independent. This effect arises because the RO_2 re-dissociation is much faster than reaction to products, so that the R and RO_2 is at equilibrium on the reaction timescale. This lack of pressure independence was essentially maintained when R5 and R6 were coupled.

Table SI2. The overall rate coefficient for product formation from R5 and R6 is given be the smallest eigenvalue of the system: 1,6 shift and 1,5-shift. Case II shown but Case I also showed no pressure dependence.

T/K	$[N_2]$/cm^{-3}	1,6-shift/s^{-1}	1,5-shift/s^{-1}	*T*/K	$[N_2]$/cm^{-3}	1,6-shift/s^{-1}	1,5-shift/s^{-1}
250	1.93E+18	4.94E-05	5.80E-07	350	1.38E+18	8.87E-01	5.64E-02
250	3.86E+18	4.94E-05	5.80E-07	350	2.76E+18	8.87E-01	5.64E-02
250	3.86E+19	4.94E-05	5.80E-07	350	2.76E+19	8.87E-01	5.64E-02
250	1.93E+20	4.94E-05	5.80E-07	350	1.38E+20	8.87E-01	5.64E-02
250	1.93E+21	4.94E-05	5.80E-07	350	1.38E+21	8.87E-01	5.64E-02
250	3.86E+22	4.94E-05	5.80E-07	350	2.76E+22	8.87E-01	5.64E-02
300	1.61E+18	1.56E-02	4.76E-04	400	1.21E+18	1.71E+01	1.99E+00
300	3.22E+18	1.56E-02	4.76E-04	400	2.41E+18	1.71E+01	1.99E+00
300	3.22E+19	1.56E-02	4.76E-04	400	2.41E+19	1.71E+01	1.99E+00
300	1.61E+20	1.56E-02	4.76E-04	400	1.21E+20	1.71E+01	1.99E+00
300	1.61E+21	1.56E-02	4.76E-04	400	1.21E+21	1.71E+01	1.99E+00
300	3.22E+22	1.56E-02	4.76E-04	400	2.41E+22	1.71E+01	1.99E+00
450	1.07E+18	1.17E+02	3.03E+01	550	8.78E+17	1.64E+02	2.76E+02
450	2.15E+18	1.17E+02	3.03E+01	550	1.76E+18	1.64E+02	2.76E+02
450	2.15E+19	1.17E+02	3.03E+01	550	1.76E+19	1.64E+02	2.76E+02
450	1.07E+20	1.17E+02	3.03E+01	550	8.78E+19	1.64E+02	2.76E+02
450	1.07E+21	1.17E+02	3.03E+01	550	8.78E+20	1.64E+02	2.76E+02
450	2.15E+22	1.17E+02	3.03E+01	550	1.76E+22	1.64E+02	2.76E+02
500	9.66E+17	1.93E+02	1.75E+02	600	8.05E+17	1.30E+02	2.46E+02
500	1.93E+18	1.93E+02	1.76E+02	600	1.61E+18	1.30E+02	2.46E+02
500	1.93E+19	1.93E+02	1.76E+02	600	1.61E+19	1.30E+02	2.46E+02
500	9.66E+19	1.93E+02	1.76E+02	600	8.05E+19	1.30E+02	2.46E+02
500	9.66E+20	1.93E+02	1.76E+02	600	8.05E+20	1.30E+02	2.46E+02
500	1.93E+22	1.93E+02	1.76E+02	600	1.61E+22	1.30E+02	2.46E+02

As the recycled OH is formed either as a direct product, R5, or via subsequent reactions, R6, it will form independent of total pressure. This lack of pressure dependence at all temperatures means that it is valid to use the pressure independent parameters proposed by Peeters *et al.* for LIM1[1] as the framework in our data analysis.

The tunnelling factor for R6 is especially important in promoting this reaction at 298 K. The tunnelling factors for R6 and R5 were calculated by Peeters *et al.* [1] and is shown in Figure SI3. While it is acknowledged that calculating tunnelling factors has significant uncertainty, this should not significantly affect our analysis as tunnelling at the temperature of our experimental is sufficiently high that the tunnelling factors are converging towards one. The accuracy of the tunnelling factors does affect our values extrapolated to 298 K. The reliability of the tunnelling factors is difficult to access, but we note that our extrapolated value of *k*(bulk) is in line with the recent value from Novelli et al.,[11] see Figure SI 6.

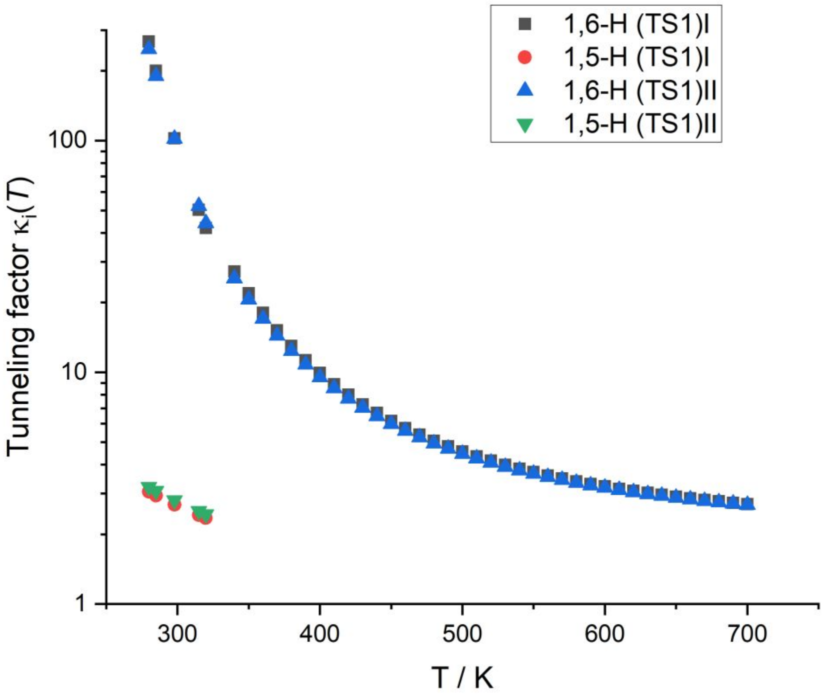

Figure SI3. The temperature dependence of the tunnelling factor as calculated by Peeters *et al.* [1] for R5 and R6.

b) Simulations of the branching ratio between HPALD and diHPCARP

Reaction of Z,Z'-OH-allyl with O_2, R7, branches to form either HPALD (7a) or diHPCARP (7b). Peeters *et al.* calculated the surface for this reaction but did not explore the kinetics. We have recalculated these surfaces of Peeters *et al.* and then carried out master equation analysis with the MESMER code.[9] These reactions that lead to HPALD and diHPCARP are independent, so the calculated rate coefficients (and branching ratio) are not absolute. The aim of these calculations is to determine how the branching ratio changes with temperature in order to extrapolate our results at high temperature to 298 K; we only need to know the relative change in k_{7a} and k_{7b} with temperature.

Overall, both oxygen addition reactions are characterised by rate coefficients that decrease with increased temperature, but the diHPCARP reaction, R7b, exhibits a slightly stronger negative temperature dependence. Our calculated $BF_{\mathrm{HPALD}}(T)$ is shown in Figure SI4, where our k_{7a} and k_{7b} have been scaled in order to yield the result of Crounse et al. [12] assuming the LIM1 model:

$$BF_{\mathrm{HPALD}}(T) = 0.234 + 0.00093\times\exp(T/111.1) \qquad \text{S-E3}$$

The average temperature of our experiments is 509 K, see Table SM1.Therefore BF_{HPALD} will be approximately 0.3 greater at our temperatures compared to 298 K. In scenarios 5, 9, 13 15 and 17, the *BF* was explored by imposing S-E3, where it was either fixed or adjusted via a scaling factor, S-E3 × BF_{scaling}.

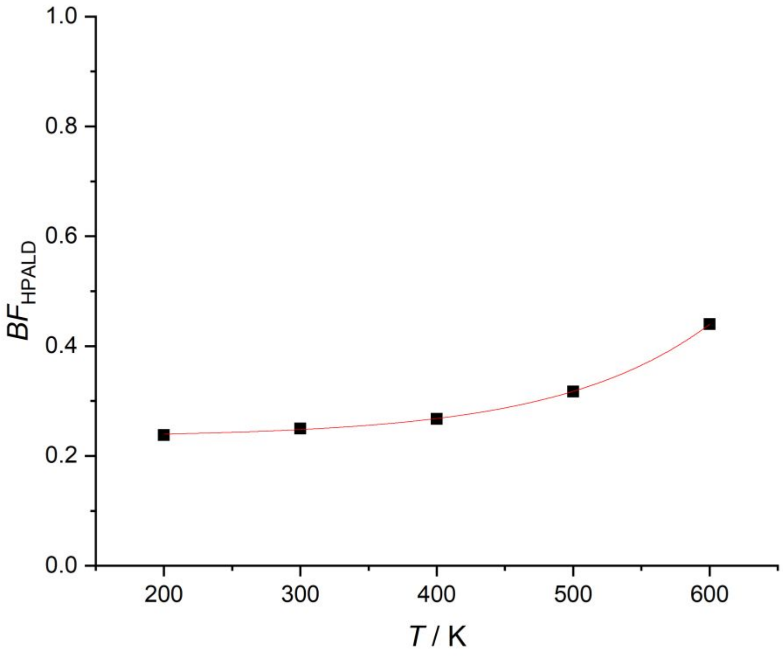

Figure SI4 – Calculated temperature dependence of BF_{HPALD} scaled to be equal to Crounse *et al.*[12] at 298 K assuming LIM1.

In general, our results confirm that *BF* is less than 0.40 and probably smaller when its temperature dependence is taking into account. Our experiments directly probe OH, so reaction R7b is measured. It is assumed that HPALD is the other channel of R7 and this channel does not decompose to OH.

5. Data analysis (MATLAB)

Data analysis was carried out globally, simultaneously fitting parameters to the 94 OH time dependent traces.[13] This approach is required as the OH trace data are described by many rate coefficients and one trace alone will not guarantee a consistent and reliable extraction of temperature-dependent information. Global analysis is a technique that takes advantage of the relationships that exist in the data to better describe and identify the parameters of the system.

LIM1 is a fundamental description of the system, where *ab initio* structure calculations were undertaken to map out the potential energy surface of the reaction (the mechanism) and reaction rate theory was employed to calculate the rate coefficients.

In our analysis, LIM1 is the starting mechanism in our data analysis seeing as its network of reactions have been verified via *ab initio* calculation, and its rate coefficient parameters calculated via reaction rate theory. The parameters (see colours in Table SI1) in of the LIM1 mechanism (plus *BF*) are explored via scenarios leading to the most flexible scenario, 14, where all the important parameters of LIM1 are adjusted. All these scenarios (1-14) are nearly equal good fits to the data (based on CHISQ) and predict a similar effective rate coefficients of the system - *k*(bulk), see Figure 2 – which means that all these scenarios (1-14) are satisfactory descriptions for atmospheric modelling.

The data analysis involves solving the LIM1 reaction scheme and adjusting the selected rate coefficients, see scenarios, in order to provide a best fit to all the OH trace data. The software package MATLAB R2016 [14] is capable of carrying out this data analysis, but requires a script to define LIM1 and adjust / impose constraints on the selected parameters during the global procedure. The ordinary differential equations of LIM1 were numerically integrated for the experimental conditions (T, [isoprene], [O_2], k_{loss}) of each one of the 94 traces with the aid of the MATLAB ODE suite.[15] Floatable parameters were adjusted following the Trust Region Reflective Algorithm.[16] The objective function was defined as the sum of squared residuals (χ^2) calculated from a comparison between experimental measurements and their corresponding numerical simulation. Each trace is appropriately weighted using the χ^2 from fitting it individually using a flexible function, a bi-exponential. This individual fit χ^2 represents the best fit, so that in the global analysis the best value for χ^2 divided by the number of traces, n_{traces}, is 1.0. From Table 1 it can be seen that χ^2/n_{traces} is within ~20% of 1.0, and section 7 shows these fits. Weighting using the individual χ^2 is

normalising each trace for signal (amplitude) and the noise within the trace. The adjusted parameters are given in the scenarios together with the best-fit parameters.

In the analysis, the R7 reactions involve O_2 and their rate coefficients were not calculated in LIM1. To explore the importance of the R7 rate coefficient, fits to data were carried (via scenario 6) with k_{R7} initially floated. This did not return a define parameter. In subsequent fits, k_{R7} was fixed with a range of values, 10^{-21} - 10^{-10} cm^3 molecule^{-1} s^{-1}. The results are summarized in Figure SI5, where it can be seen that CHISQ reduces as k_{R7} increases, but once $k_7 \geq 10^{-13}$ cm^3 molecule s^{-1} χ^2 reaches its minimum. This means that k_7 is not defined, except that it requires a minimum value. In our analysis k_7 was set > 4×10^{-14} cm^3 molecule^{-1} s^{-1}.

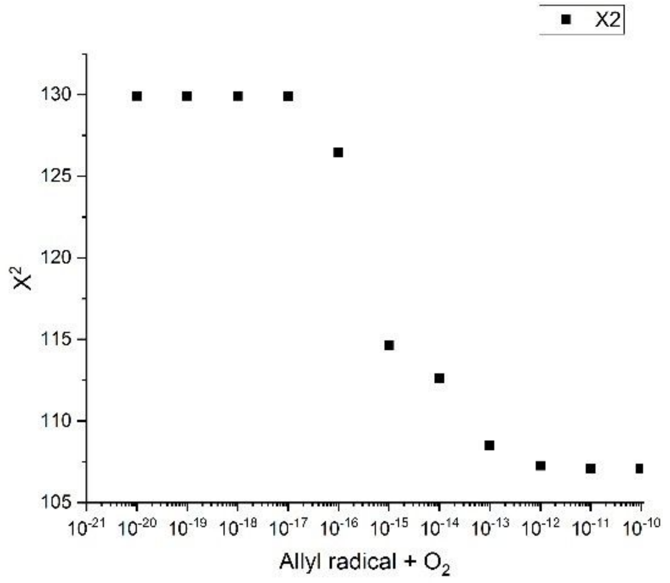

Figure SI5 The dependence of the value of the fit parameter, χ^2 vs k_7.

SCENARIOS

The LIM1 mechanism (and Caltech) is explored by starting with the basic LIM1 mechanism and is explored over a range of scenarios, where the following parameters have been floated: A_3 via both $A_{3,Z\text{-}\delta\text{-}RO2}$ and $A_{3,other\text{-}RO2}$; barriers to products either via linking the 1,6 and 1,5 barriers or floating them unlinked; and the binding energy of the RO_2. All the energies

are given as the change from the values in LIM1. The branching ratio of R7, *BF,* was tested with a range of models. For scenarios 1, 6, 8 and 9, *BF* was assigned using the equation:

$$BF_{HPALD} = 4.3160 \times 10^{-7} \times \left(\frac{-1160 + Ea_{1-6-H\ shift}}{R}\right)^{2} - 7.8937 \times 10^{-3} \times \frac{-1160 + Ea_{1-6-H\ shift}}{R} + 36.187 \quad \text{S-E4}$$

where $Ea_{1\text{-}6\text{-H shift}}$ is the value from case 1. In scenarios 3, 5, 7, 11 and 13 the *BF* was floated as temperature dependent based on equation S-E3, i.e. S-E3 × $BF_{scaling}$. In scenarios 2, 4, 10, 12 and 14 *BR* was floated as temperature independent.

Table SI3 - Results from different scenarios. All quoted errors 2σ.

Scenario and Description	$A_{3z}/A_{3z,LIM1}$	$A_{3,other}/A_{3,other,LIM1}$	$BF_{scaling}$ *BF(298 K)*	Difference in $E_{1,5}$ from LIM1	Difference in $E_{1,6}$ from LIM1	χ^2	$t_{1/2}$/s at 298 K
**1.** LIM1 with only A_3 adjusted. All A_3 linked. *BR* from eqn SE4.	2.9 ± 1.1[a]		0.25			108.0	85
**2.** As **1** but with *BR* floated independent of temperature.	4.0 ± 1.9		0.38 ± 0.02			107.2	85
**3.** As **1** but *BR* floated via eqn SE3.[b]	3.8 ± 1.5		0.97 ± 0.05[b]<br>0.24 ± 0.02[b]			107.2	85
**4.** As **2** but A_3 values allowed to float separately.	4.1 ± 0.3	4.0 ± 1.4	0.38 ± 0.02			107.2	85 ± 14
**5.** As **3** but A_3 values allowed to float separately.	4.1 ± 0.3	4.0 ± 1.4	1.00 ± 0.07[b]<br>0.25 ± 0.01[b]			107.7	98 ± 2
**6.** A3 allowed to float separately, *BF* fixed via eqn SE4, $E_{1,5}$ and $E_{1,6}$ linked and floated.	6.2 ± 0.7	4.4 ± 2.1	0.33	1.1 ± 0.2[c]		106.6	85 ± 18
**7.** As **6** but *BF* fixed to eqn SE3, i.e. scaling factor is 1.00	4.7 ± 1.3	4.8 ± 1.3	1.00<br>0.25	0.6 ± 0.8		107.7	107 ± 19
**8.** Allow barriers to float independently. A_3 linked. *BF* from eqn SE4.	5.7 ± 3.2		0.19	6.0 ± 1.7	-1.0 ± 0.3	106.0	76 ± 26
**9.** As **8** but A_3 allowed to float separately.	5.7 ± 0.7	5.7 ± 2.6	0.19	6.2 ± 2.2	-1.0 ± 0.6	106.0	75 ± 16
**10.** As **9** but *BF* allowed to float as temperature independent parameter.	9.1 ± 5.3	4.7 ± 2.1	0.47 ± 0.09<br>0.21 ± 0.04	3.8 ± 1.7	2.0 ± 2.9	106.3	95 ± 35
**11.** As **9** but *BF* allowed to float via eqn SE3.[b]	8.8 ± 4.7	5.2 ± 2.4	0.12 ± 0.02	5.4 ± 2.0	1.6 ± 2.6	106.8	95 ± 24
**12.** As **6** but *BF* allowed to float as a temperature independent value.	13.4 ± 4.4	4.2 ± 0.4	0.21 ± 0.04	3.7 ± 1.3		106.7	93 ± 6
**13.** As **6** but *BF* allowed to float via eqn SE3.[b]	18.6 ± 5.6	4.2 ± 0.5	0.44 ± 0.08<br>0.11 ± 0.02	5.0 ± 1.3		106.9	106 ± 6
**14.** All parameters floated including R + O_2 binding energy (-0.3 ± 2.0). *BF* floated as temperature independent parameter.	11.7 ± 8.0	4.8 ± 2.4	0.19 ± 0.04	4.5 ± 1.9	2.8 ± 2.8	106.6	99 ± 71
**15.** Caltech model	34.3 ± 5.9			3.9 ± 0.2		140.2	533 ±23
**16.** As **15** but A parameters split	8.4 ± 1.4	20.1 ± 2.6		2.7 ± 0.2		138.9	664 ± 28

a – When only a single value is given the A factors are the same for all R + O_2 reactions.

b – *BF* calculated from equation SE3.

c – Both barriers have been altered in unison.

SUMMARY OF SCENARIOS

With the exception of the Caltech mechanism, these many scenarios (1- 14) all provide a near equal good fit to the data. With respect to the kinetics of the system, it does not matter too much which model is chosen as they all lead to similar species profiles for the formation of products: MVK/MAC and diHPCARP/HPALD. This point is demonstrated in Figure SI6, where it can be seen that *k*(bulk) at 298 K is essentially the same for all the LIM1 (scenario 1) and LIM1-Leeds models. The Caltech model produces a much smaller *k*(bulk) lifetime, but this scenario fit is significantly worse and hence inconsistent with our data. In Figure SI6, the literature *k*(bulk) results from Novelli et al., [11] MCM 3.3.1 [17] and Caltech [18] have been added to the scenarios plot, where it can be seen that our result is in reasonable agreement with Novelli et al.[11] There is more uncertainty in the branching ratio for R7, *BF*, see Figure SI7. It is clearly less than 0.4 and based on the temperature dependence of *BF*, see Figure SI4, is more likely to be ≤0.25, and most likely to be lower.

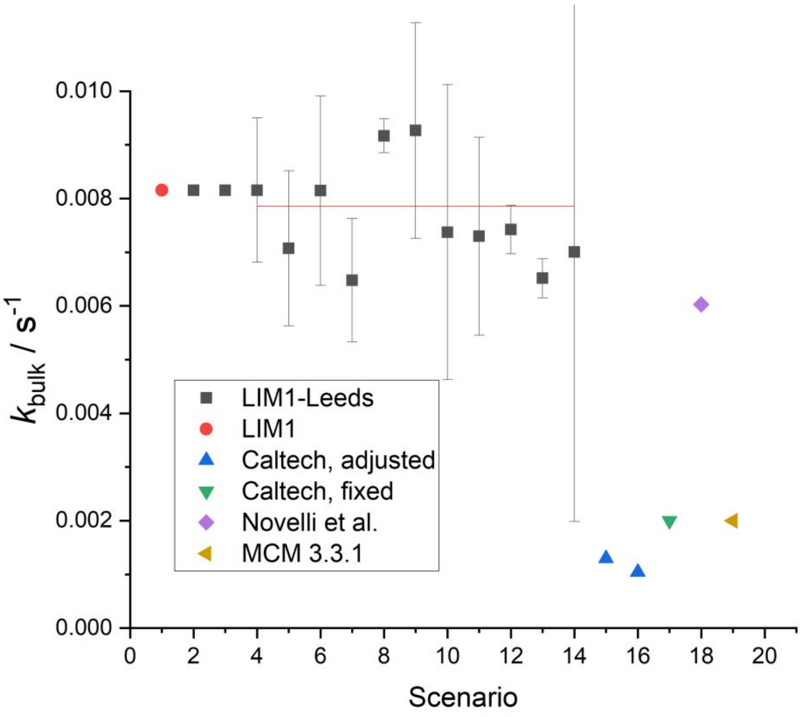

Figure SI6 Plot of the *k*(bulk) for each scenario, plus the literature values from Novelli et al.,[11] MCM 3.3.1 [17] and the Caltech model.[18]

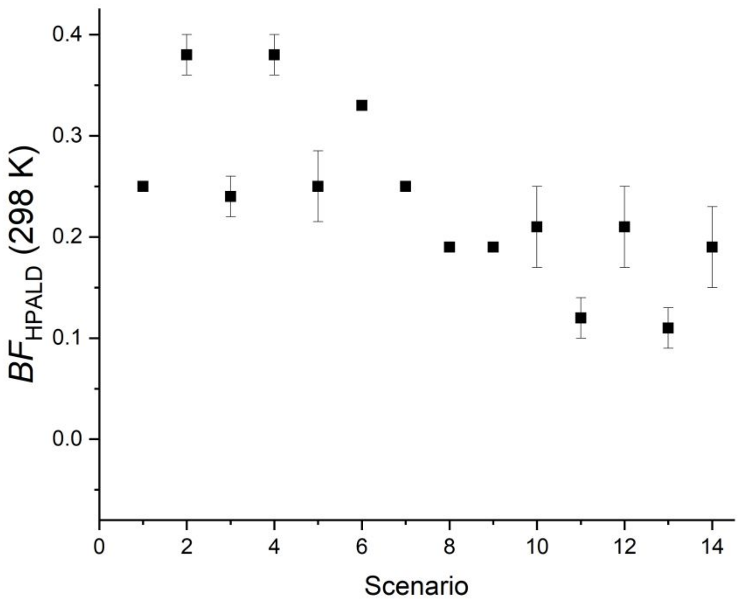

Figure SI7 Plot of the branching ratio for R7, *BR*, at 298 K for the various scenarios.

All the LIM1-LIM1-Leeds scenarios, 1-14, return defined parameters, where the errors increase as more parameters are floated. However, large parameter errors do not necessarily lead to equally large errors in the observables. To make this point a number of Monte-Carlo (MC) simulations have been untaken using dependent multivariate distributions (via the correlation matrix from the scenario fit to the data). These MC simulations allowed the half-life to be determined, which is used to calculate *k*(bulk), see Figure 2. It also allows the statistics on *k*(bulk) to be determined. Figure SI8 shows the species profiles for all products from the MC simulation of scenario 14. This scenario has the most floated parameters, with greatest errors, and still generates species profile with less than 30% uncertainty. It is noted that multivariate distribution sampling leads to less uncertainty than if MC sampled according to just the errors in the parameters, i.e. the correlations in the parameters serve to better define the product distributions.

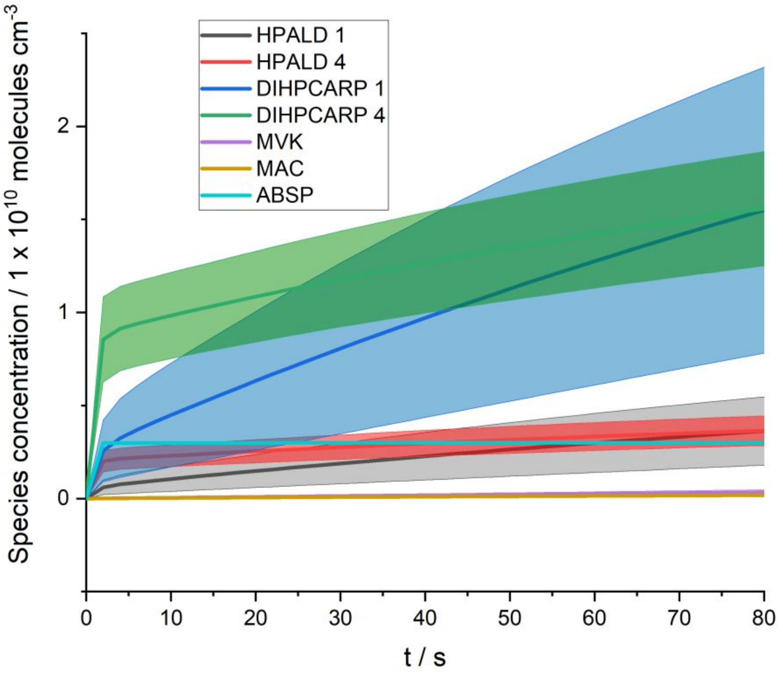

Figure SI8 Monte-Carlo simulations of the result from scenario 11 at 295 K. $[OH]_0$ was equal to 10^{11} molecule cm^{-3} and [Isoprene], $[O_2]$ sufficiently large that the result is independent of these concentrations. Any OH product was not allowed to recycle, so that at long times the sum of all the products is equal to $[OH]_0$. Compared to Figure 3, this shows the short time behaviour, where it can be seen that diHPCARP 4 forms quicker than diHPCARP 1.

6. Further MCM Modelling

In the main text, Figure 5 shows further MCM 0-D box modelling (chemistry only) of the OP3 Borneo campaign [19, 20], where it is shown that enhanced photolysis of the di-HPCARP decomposition product, dihydroperoxide carbonyl, DHP, can significantly enhance [OH]. This is further explored in Figure SI9. Increasing the DHP photolysis rate by a factor of 10 and 200 produces significant increases in [OH] compared to the MCM photolysis DHP photolysis rate, LIM1-Leeds model. However, j(DHP)×1000 produces essentially the same [OH] as j(DHP)×200, so there is a limit to the increase in [OH] via enhanced DHP photolysis.

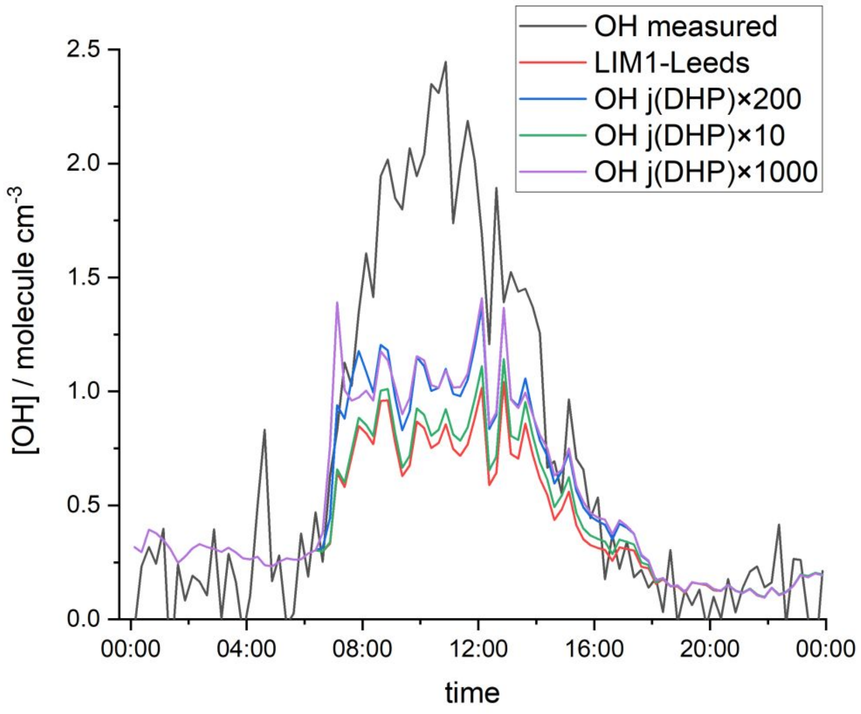

Figure SI9 The effect of enhanced DHP photolysis on [OH]. LIM1-Leeds has DHP photolysis equal to the MCM, i.e. j(DHP)×1.

7. *Graphical Summary of the Fits to the Experimental Data*

1

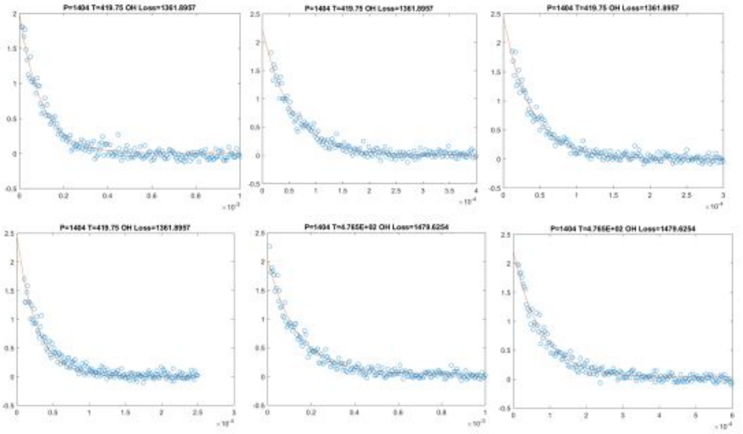

2

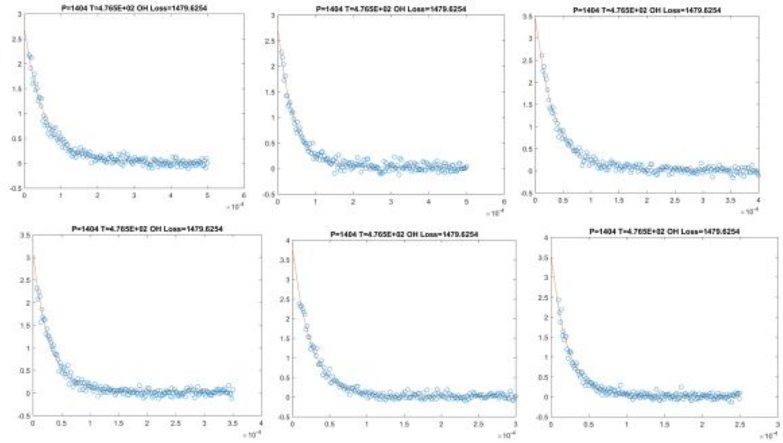

3

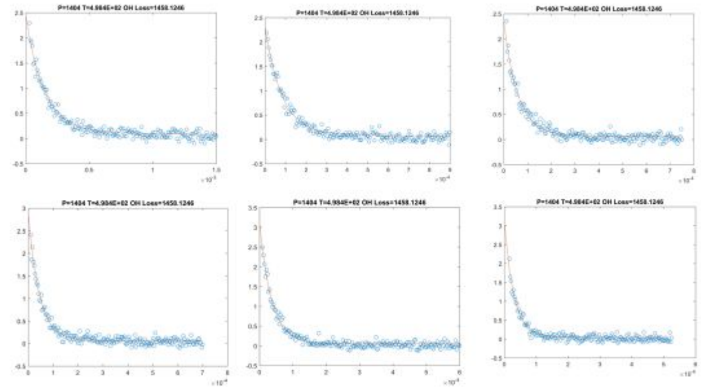

4

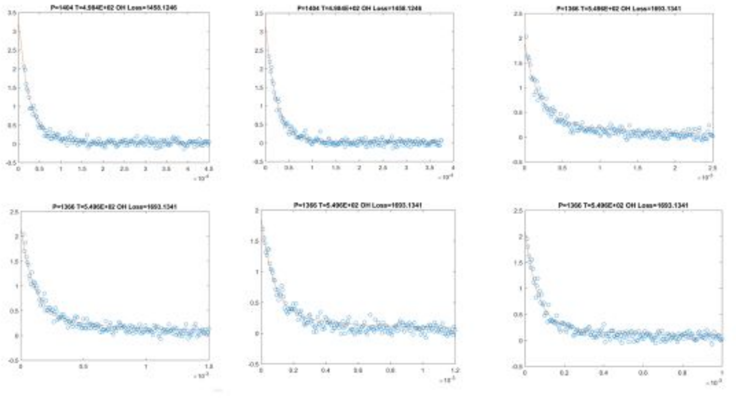

5

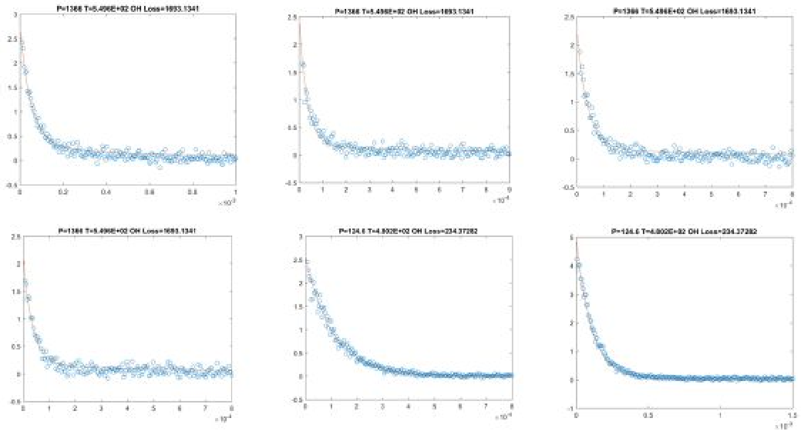

6

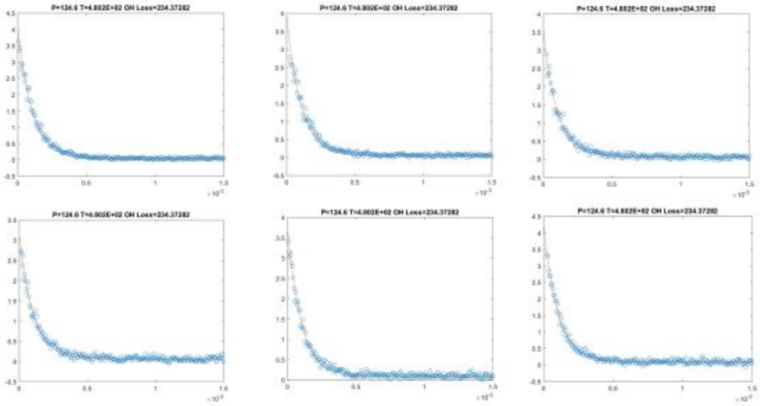

7

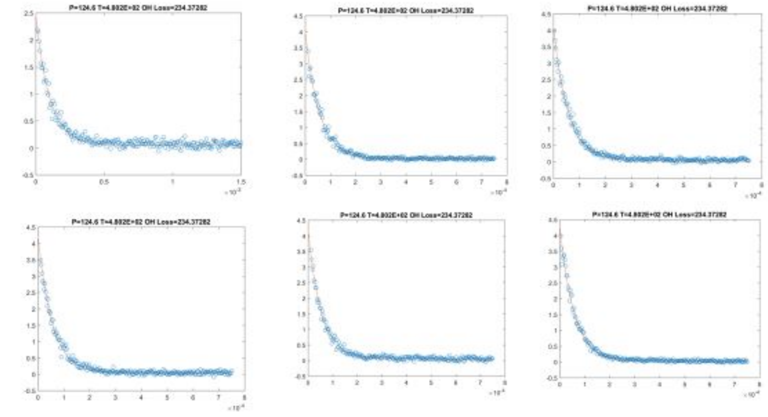
P=124.6 T=4.802E+02 OH Loss=234.37282
P=124.6 T=4.802E+02 OH Loss=234.37282
P=124.6 T=4.802E+02 OH Loss=234.37282
P=124.6 T=4.802E+02 OH Loss=234.37282
P=124.6 T=4.802E+02 OH Loss=234.37282
P=124.6 T=4.802E+02 OH Loss=234.37282

8

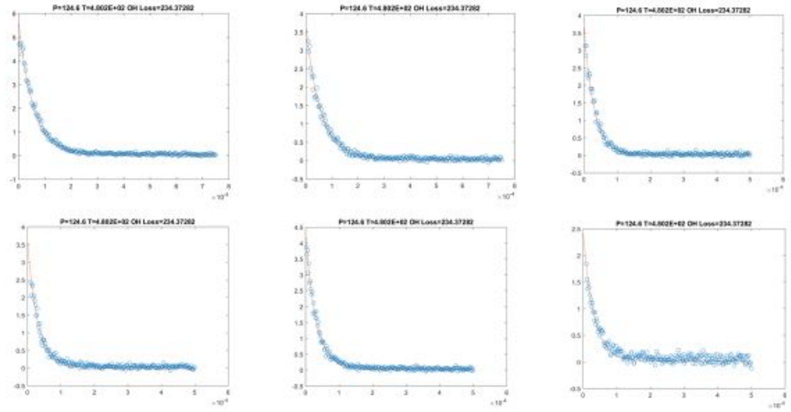
P=124.6 T=4.802E+02 OH Loss=234.37282
P=124.6 T=4.802E+02 OH Loss=234.37282
P=124.6 T=4.802E+02 OH Loss=234.37282
P=124.6 T=4.802E+02 OH Loss=234.37282
P=124.6 T=4.802E+02 OH Loss=234.37282
P=124.6 T=4.802E+02 OH Loss=234.37282

9

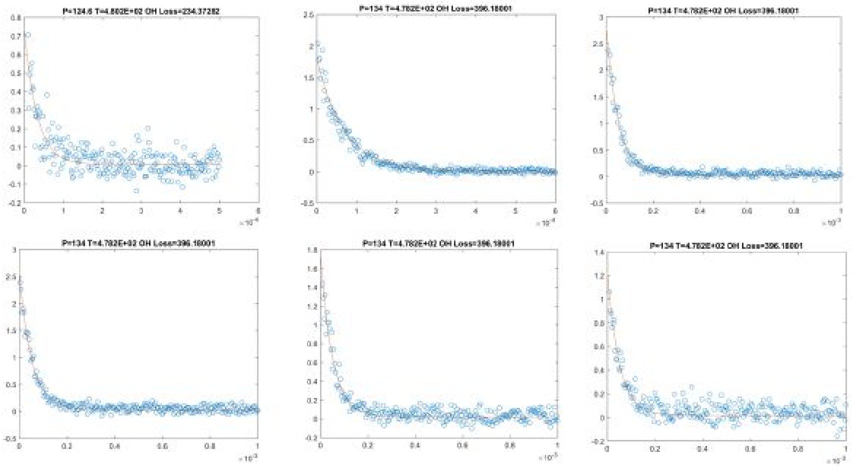
P=124.6 T=4.802E+02 OH Loss=234.37282
P=134 T=4.782E+02 OH Loss=396.18001
P=134 T=4.782E+02 OH Loss=396.18001
P=134 T=4.782E+02 OH Loss=396.18001
P=134 T=4.782E+02 OH Loss=396.18001
P=134 T=4.782E+02 OH Loss=396.18001

10

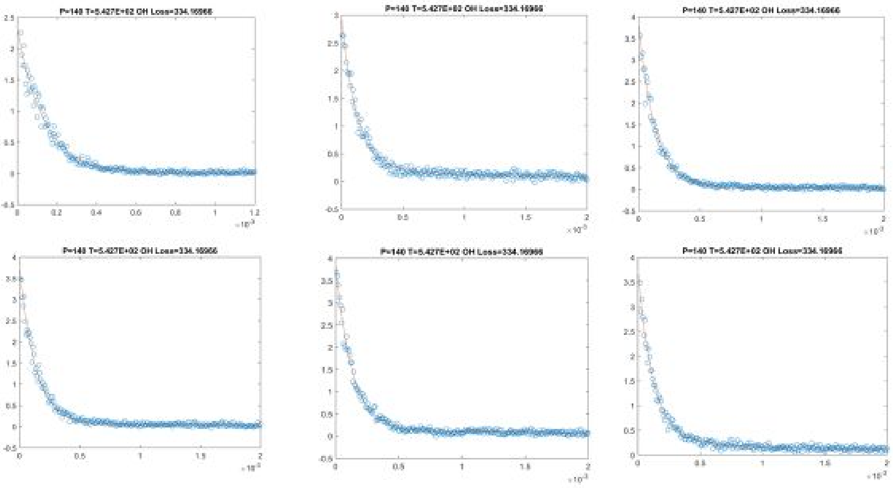
P=140 T=5.427E+02 OH Loss=334.16966
P=140 T=5.427E+02 OH Loss=334.16966
P=140 T=5.427E+02 OH Loss=334.16966
P=140 T=5.427E+02 OH Loss=334.16966
P=140 T=5.427E+02 OH Loss=334.16966
P=140 T=5.427E+02 OH Loss=334.16966

11

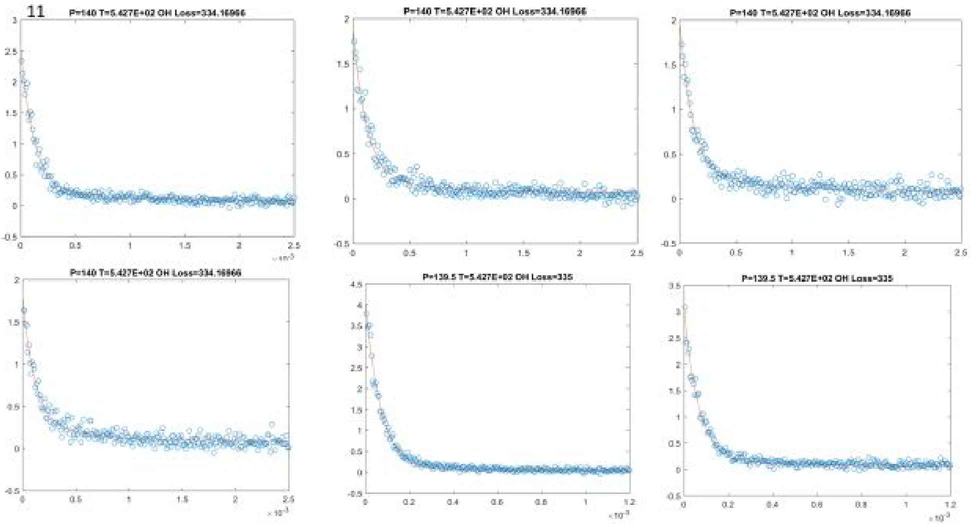
P=140 T=5.427E+02 OH Loss=334.16966
P=140 T=5.427E+02 OH Loss=334.16966
P=140 T=5.427E+02 OH Loss=334.16966
P=140 T=5.427E+02 OH Loss=334.16966
P=139.5 T=5.427E+02 OH Loss=335
P=139.5 T=5.427E+02 OH Loss=335

12

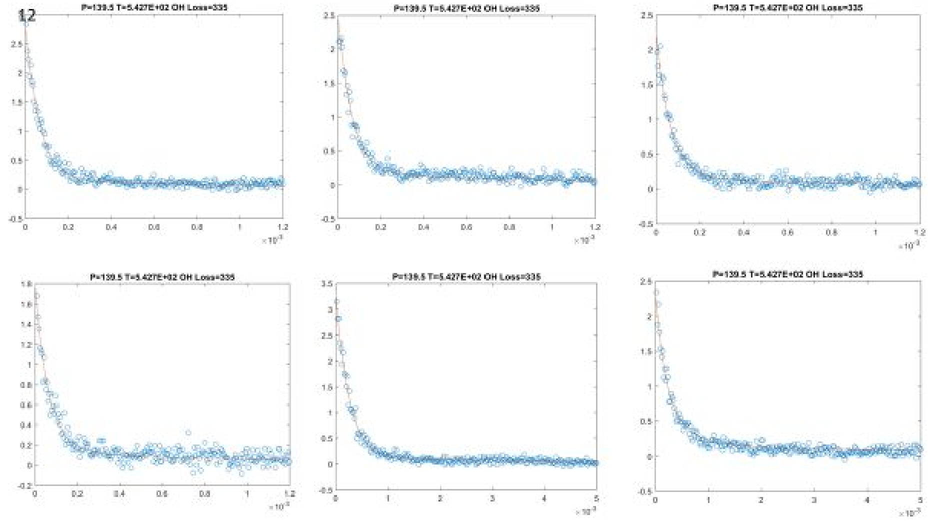
P=139.5 T=5.427E+02 OH Loss=335
P=139.5 T=5.427E+02 OH Loss=335
P=139.5 T=5.427E+02 OH Loss=335
P=139.5 T=5.427E+02 OH Loss=335
P=139.5 T=5.427E+02 OH Loss=335
P=139.5 T=5.427E+02 OH Loss=335

13

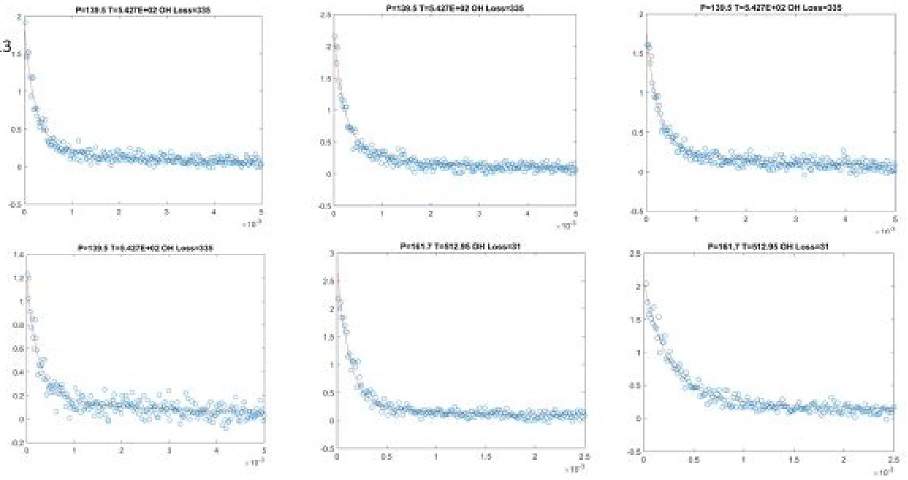
P=139.5 T=5.427E+02 OH Loss=335
P=139.5 T=5.427E+02 OH Loss=335
P=139.5 T=5.427E+02 OH Loss=335
P=139.5 T=5.427E+02 OH Loss=335
P=161.7 T=512.95 OH Loss=31
P=161.7 T=512.95 OH Loss=31

14

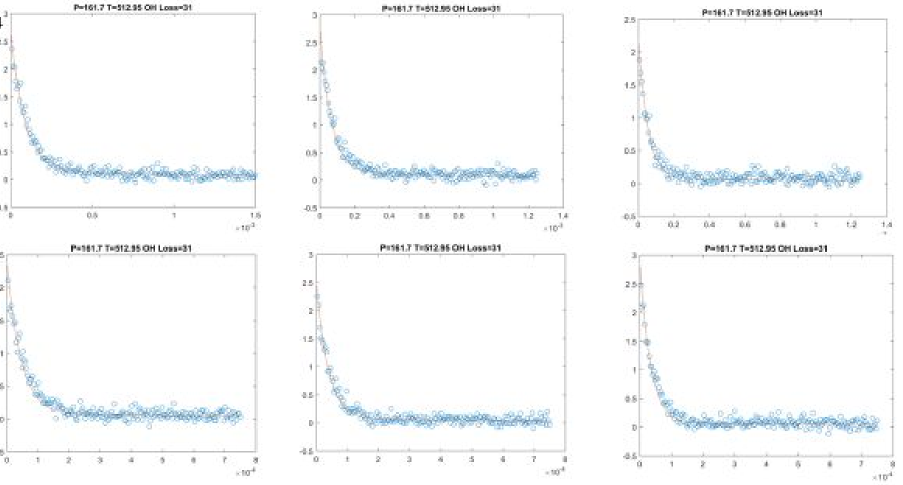
P=161.7 T=512.95 OH Loss=31
P=161.7 T=512.95 OH Loss=31
P=161.7 T=512.95 OH Loss=31
P=161.7 T=512.95 OH Loss=31
P=161.7 T=512.95 OH Loss=31
P=161.7 T=512.95 OH Loss=31

15

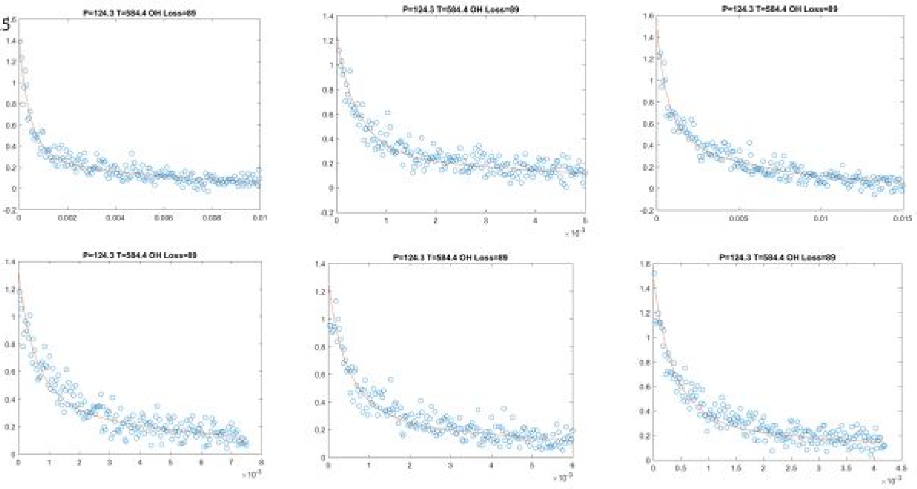
P=124.3 T=584.4 OH Loss=89
P=124.3 T=584.4 OH Loss=89
P=124.3 T=584.4 OH Loss=89
P=124.3 T=584.4 OH Loss=89
P=124.3 T=584.4 OH Loss=89
P=124.3 T=584.4 OH Loss=89

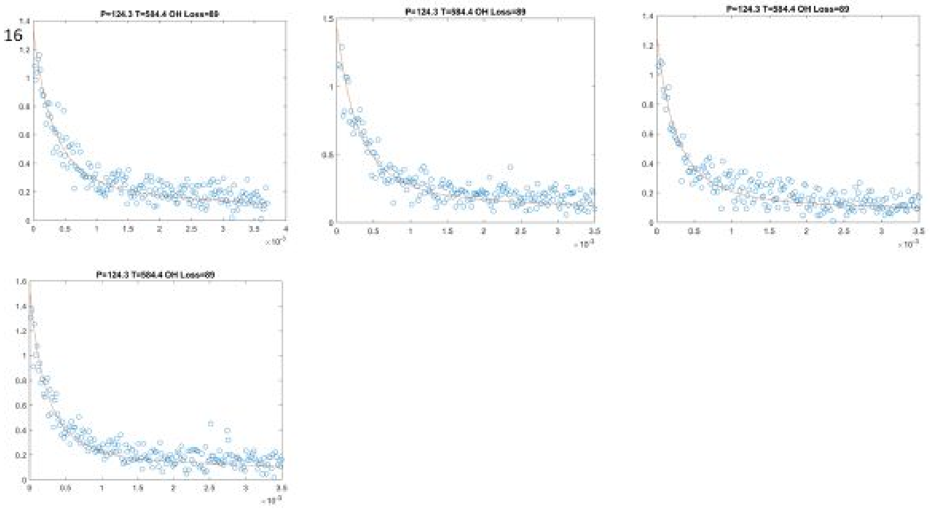

8. References

1. Peeters J, Muller JF, Stavrakou T, Nguyen VS. Hydroxyl Radical Recycling in Isoprene Oxidation Driven by Hydrogen Bonding and Hydrogen Tunneling: The Upgraded LIM1 Mechanism. *J Phys Chem A* 2014, **118**(38)**:** 8625-8643.

2. Medeiros DJ, Blitz MA, James L, Speak TH, Seakins PW. Kinetics of the Reaction of OH with Isoprene over a Wide Range of Temperature and Pressure Including Direct Observation of Equilibrium with the OH Adducts. *The Journal of Physical Chemistry A* 2018, **122**(37)**:** 7239-7255.

3. Glowacki DR, Lockhart J, Blitz MA, Klippenstein SJ, Pilling MJ, Robertson SH*, et al.* Interception of Excited Vibrational Quantum States by O-2 in Atmospheric Association Reactions. *Science* 2012, **337**(6098)**:** 1066-1069.

4. Onel L, Blitz MA, Seakins PW. Direct Determination of the Rate Coefficient for the Reaction of OH Radicals with Monoethanol Amine (MEA) from 296 to 510 K. *J Phys Chem Lett* 2012, **3**(7)**:** 853-856.

5. Speak TH, Blitz MA, Stone D, Seakins PW. A New Instrument for Time Resolved Measurement of HO2 Radicals. *Atmos Meas Tech Discuss* 2019, **2019:** 1-30.

6. Creasey DJ, HalfordMaw PA, Heard DE, Pilling MJ, Whitaker BJ. Implementation and initial deployment of a field instrument for measurement of OH and HO2 in the troposphere by laser-induced fluorescence. *J Chem Soc-Faraday Trans* 1997, **93**(16)**:** 2907-2913.

7. Stone D, Blitz M, Ingham T, Onel L, Medeiros DJ, Seakins PW. An instrument to measure fast gas phase radical kinetics at high temperatures and pressures. *Review of Scientific Instruments* 2016, **87**(5)**:** 054102.

8. Atkinson R, Baulch DL, Cox RA, Crowley JN, Hampson RF, Hynes RG*, et al.* Evaluated kinetic and photochemical data for atmospheric chemistry: Volume I - gas phase reactions of O-x, HOx, NOx and SOx species. *Atmos Chem Phys* 2004, **4:** 1461-1738.

9. Glowacki DR, Liang CH, Morley C, Pilling MJ, Robertson SH. MESMER: An Open-Source Master Equation Solver for Multi-Energy Well Reactions. *J Phys Chem A* 2012, **116**(38)**:** 9545-9560.

10. Carr SA, Glowacki DR, Liang CH, Baeza-Romero MT, Blitz MA, Pilling MJ*, et al.* Experimental and Modeling Studies of the Pressure and Temperature Dependences of the Kinetics and the OH Yields in the Acetyl + O-2 Reaction. *J Phys Chem A* 2011, **115**(6)**:** 1069-1085.

11. Novelli A, Vereecken L, Bohn B, Dorn HP, Gkatzelis GI, Hofzumahaus A*, et al.* Importance of isomerization reactions for OH radical regeneration from the photo-oxidation of isoprene investigated in the atmospheric simulation chamber SAPHIR. *Atmos Chem Phys* 2020, **20**(6)**:** 3333-3355.

12. Crounse JD, Paulot F, Kjaergaard HG, Wennberg PO. Peroxy radical isomerization in the oxidation of isoprene. *Phys Chem Chem Phys* 2011, **13**(30)**:** 13607-13613.

13. Beechem JM, Knutson JR, Brand L. Global Analysis of Multiple Dye Fluorescence Anisotropy Experiments on Proteins. *Biochem Soc Trans* 1986, **14**(5)**:** 832-835.

14. *MATLAB and Optimization Toolbox Release R2016a*. Natwick : Math Works Inc., 2016.

15. Shampine LF, Reichelt MW. The MATLAB ODE Suite. *SIAM J Sci Comput* 1997, **18**(1)**:** 1-22.

16. Moré JJ, Sorensen DC. Computing a Trust Region Step. *SIAM J Sci Stat Comput* 1983, **4**(3)**:** 553-572.

17. Jenkin ME, Young JC, Rickard AR. The MCM v3.3.1 degradation scheme for isoprene. *Atmos Chem Phys* 2015, **15**(20)**:** 11433-11459.

18. Wennberg PO, Bates KH, Crounse JD, Dodson LG, McVay RC, Mertens LA*, et al.* Gas-Phase Reactions of Isoprene and Its Major Oxidation Products. *Chem Rev* 2018, **118**(7)**:** 3337-3390.

19. Stone D, Evans MJ, Edwards PM, Commane R, Ingham T, Rickard AR*, et al.* Isoprene oxidation mechanisms: measurements and modelling of OH and HO_2 over a South-East Asian tropical rainforest during the OP3 field campaign. *Atmos Chem Phys* 2011, **11**(13)**:** 6749-6771.

20. Edwards PM, Evans MJ, Furneaux KL, Hopkins J, Ingham T, Jones C*, et al.* OH reactivity in a South East Asian tropical rainforest during the Oxidant and Particle Photochemical Processes (OP3) project. *Atmos Chem Phys* 2013, **13**(18)**:** 9497-9514.
